# Advances and Challenges in Smart Packaging Technologies for the Food Industry: Trends, Applications, and Sustainability Considerations

**DOI:** 10.3390/foods14244347

**Published:** 2025-12-17

**Authors:** Mădălina Alexandra Davidescu, Claudia Pânzaru, Bianca Maria Mădescu, Ioana Poroșnicu, Cristina Simeanu, Alexandru Usturoi, Mădălina Matei, Marius Gheorghe Doliș

**Affiliations:** “Ion Ionescu de la Brad” Iasi University of Life Sciences, 3 Mihail Sadoveanu Alley, 700490 Iasi, Romania; madalina.davidescu@iuls.ro (M.A.D.); bianca.madescu@iuls.ro (B.M.M.); ioana.porosnicu@yahoo.com (I.P.); cristina.simeanu@iuls.ro (C.S.); alexandru.usturoi@iuls.ro (A.U.); madalina.matei@iuls.ro (M.M.); marius.dolis@iuls.ro (M.G.D.)

**Keywords:** food safety, intelligent packaging, smart packaging, nanomaterials, quality sensors, sustainability

## Abstract

Recent advancements in food packaging have transitioned from passive containment toward innovative smart systems that integrate active and intelligent functionalities to improve product preservation, safety, and consumer interaction. This review examines the evolution of these technologies, focusing on biodegradable polymers and nanomaterial-enhanced substrates that combine environmental sustainability with superior barriers and antimicrobial performance. Developments in embedded sensing systems, including chemical, temperature, and humidity sensors, enable the continuous monitoring of food quality and environmental conditions, supporting extended shelf-life and early contamination detection. Intelligent packaging further incorporates indicators, sensors, and data carriers that enhance transparency and traceability across supply chains. These systems are often connected through blockchain and Internet of Things (IoT) platforms for real-time data analysis. The review also addresses consumer engagement via interactive labels and personalized nutritional feedback, along with the economic, behavioral, and regulatory aspects influencing large-scale adoption. Life cycle assessments are analyzed to evaluate trade-offs between enhanced functionality and environmental impact, emphasizing recyclability and end-of-life strategies within circular economy frameworks. Finally, the article discusses current technical challenges while highlighting emerging trends such as AI-driven predictive analytics and IoT-enabled connectivity as key enablers of sustainable, efficient, and safe food packaging systems.

## 1. Introduction

The packaging industry is undergoing rapid transformation, driven by emerging technologies and increased consumer awareness of sustainability and food safety. There is a growing trend toward innovations that combine technological sophistication with environmental responsibility in the food sector [1,2,3]. A key focus is advanced smart packaging, defined as systems that interact with their contents or the environment to monitor and provide real-time data regarding product condition, authenticity, and safety [1,3,4]. These systems use external parameters such as humidity, temperature, and gas composition, or internal indicators such as metabolites, to monitor product quality [2,3,4,5,6].

A major catalyst for these innovations is the rising regulatory pressure and market demand for eco-friendly solutions [7]. In developed economies, policies promoting sustainable materials create opportunities for manufacturers who combine environmental goals with modern, functional design. This reflects dual movement: the ecological reform of packaging materials and the concurrent integration of intelligent features within them [1,4,8].

Smart packaging lies at the intersection of multiple scientific and engineering disciplines. Nanotechnology plays a central role by enabling nanocomposites and nanofibrous structures that enhance performance through selective barrier properties and reactive surfaces [3,4,5,6,9]. Electrospinning techniques used to fabricate nanofibers provide precise control over porosity and surface chemistry, not only improving protection against contamination but also enabling specialized applications, such as bacterial culture platforms [10,11]. These nanoengineered systems are increasingly recognized as essential components for achieving active and intelligent packaging functions.

The integration of biosensors into food packaging enhances the functional capabilities of the packaging system. Supply chain managers and consumers can benefit from the dynamic feedback offered by these sensors that detect spoilage markers or harmful contaminants inside the package [3,4,5,6,7,12,13]. The challenges persist, including concerns about biosensor safety, precise control over particle size in bionanocomposites, and achieving a balance between affordability and environmental compatibility. Future research must consider the trade-offs between innovation and sustainability.

Beyond laboratory development, smart packaging directly contributes to food logistics. Time–temperature indicators (TTIs) enable precise monitoring during transport, particularly in the “last mile,” where temperature fluctuations are common [14,15,16,17,18]. These systems improve inventory control, delivery coordination, and consumer transparency. Blockchain-linked packaging adds traceability and protects against counterfeiting, offering economic benefits by reducing waste and enhancing brand trust [19]. Still, scalability is limited by high integration costs and difficulties in combining electronic components with biodegradable materials.

Consumer perception also plays a significant role in determining the adoption of smart packaging technologies. Acceptance varies according to technological familiarity and the level of trust placed in regulatory oversight. While some consumers value features such as freshness indicators and origin verification, others express concerns regarding privacy when packaging systems transmit data digitally [5,7,20]. Enhancing public understanding of the lifecycle of smart packaging, from production to disposal, may help reduce these concerns. Moreover, smart systems are increasingly incorporated into food defense strategies, enabling authenticity verification and tamper detection to mitigate risks of fraud and adulteration. The integration of such intelligent functionalities with active preservation mechanisms strengthens product safety and transparency across global supply chains.

Technological classifications distinguish between active packaging, which interacts chemically or biologically to maintain quality, and intelligent packaging, which communicates product status [2,11,12,13,14,21,22]. Increasingly, these functionalities are integrated, for example, into edible films derived from fruit or vegetable sources that incorporate freshness sensors, thereby combining renewable materials with digital monitoring capabilities to reduce food waste.

Overall, smart packaging lies at a complex intersection of sustainability, technology, economics, and consumer perception. Advances in materials science, sensor design, and supply chain integration will determine how effectively food integrity evolves from the point of a passive container to an active system ensuring food integrity throughout its lifecycle [7,9,23].

Numerous studies demonstrate the growing importance of smart packaging in the food industry and examine various aspects of its development and functionality [1,2,3,4,5,7,9,10,11,12,13,14,15,16,17,18,19,20,21,22,23]. This review explores current developments and challenges in smart packaging, emphasizing how nanotechnology and biosensors can enhance food safety and sustainability. Its practical goal is to identify scalable, eco-compatible solutions that can be implemented in real-world food supply chains to reduce waste and improve product traceability. In this context, the analysis advances the field by integrating recent findings and delineating emerging research pathways that are poised to guide the future development and application of smart packaging system.

## 2. Background and Evolution of Food Packaging

The background and evolution of food packaging illustrate a progressive shift from basic protective functions to sophisticated, sustainable, and technology-driven systems developed to meet contemporary requirements for safety, quality, and consumer engagement.

### 2.1. Historical Development of Packaging Technologies

**Figure 1:**
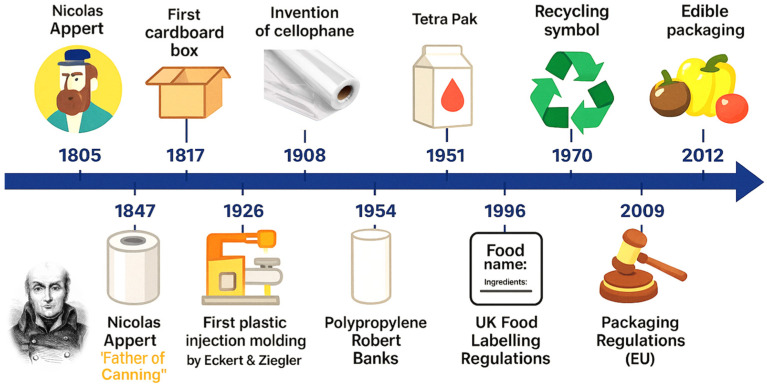
Historical evolution of food packaging.

The progression of packaging technologies in the food industry traces back to rudimentary containment methods, which served primarily as physical barriers against external contaminants. Early societies relied on naturally occurring materials such as leaves, animal skins, gourds, and woven fibers to store and transport food items. Although these organic solutions offered limited preservation capacity, they were widely available and easy to obtain. With advancements in metallurgy and ceramics, packaging evolved into more durable formats like tin containers and clay amphorae that offered better protection from moisture and pests [2,8,24,25]. During the industrial revolution, standardized manufacturing enabled widespread adoption of glass bottles, metal cans, and paper-based wraps, ushering in mass production of packaged goods with consistent quality. The emergence of plastics in the mid-20th century marked a substantial transformation [18,26,27,28]. Petroleum-based polymers were inexpensive to produce at scale while offering lightweight form factors, versatility in molding shapes, and resistance to mechanical damage (Figure 1). This era saw the rapid integration of polyethylene, polypropylene, and PET into the supply chain [29].

However, this heavy dependence on synthetic polymers generated environmental concerns due to their persistence in ecosystems. These issues later catalyzed research into biopolymers derived from renewable resources as alternatives for sustainable development. Initially, packaging functionality was mostly passive, focused on containment and occasional esthetic branding [7,17,30,31]. The conceptual shift toward active packaging caused significant changes in the mid- to late-20th century. Incorporating antimicrobial emitters or oxygen scavengers directly into film layers allowed food quality to be maintained over longer periods without bulk chemical preservatives. Active mechanisms interacted with the internal atmospheres of the package to inhibit microbial growth or oxidative degradation processes [32]. Concurrently, intelligent packaging emerged as a distinct paradigm, characterized by systems capable of communicating product condition through embedded indicators or sensors. By the late 20th century, heightened regulatory attention to public health further increased interest in dynamically monitoring food quality through the packaging itself [8,21,33]. The development of smart labels using responsive polymers, such as polyaniline-based sensing films that change color upon detecting spoilage volatiles, demonstrated how visual cues could inform consumers in real time [34]. This intersection between material science and consumer transparency laid the foundation for integrating microelectronics into food packages. The historical trajectory also reflects societal influences: evolving consumer expectations for convenience accelerated the adoption of resealable containers, while globalization demanded greater shelf life for long-distance shipping. Packaging has shifted from an ancillary aspect of food production to a strategic tool influencing supply chain logistics and brand differentiation [5,35]. Active temperature management via time–temperature indicators strengthened transport reliability, especially during critical “last mile” delivery phases. Early biodegradable solutions often struggled with mechanical robustness and barrier properties compared to petrochemical plastics. Refinement using nanocomposite structures has partially bridged this gap, fortifying biopolymer films while preserving composability profiles [36]. Alongside technological innovation, legislative frameworks evolved to reflect shifts in scientific understanding of material safety and waste management. Packaging regulations expanded beyond labeling accuracy into domains such as recyclability standards and migration limits for potential contaminants from package materials into food items [37]. Harmonization of standards across regions emerged gradually but remains inconsistent, affecting globalized deployment strategies. Intelligent packaging now interfaces with blockchain systems to generate traceability records across distribution chains. This capability builds upon the long-established foundations of product labeling, and transforms them into a robust mechanism against fraud and mislabeling by embedding digital verification at each stage of the supply chain [25,38,39]. Despite the perceived linearity from primitive containment toward modern smart systems, historical development exhibits cyclical elements. For example, the early emphasis on local organic materials resonates today as researchers return to plant starch derivatives like cassava-based smart films using anthocyanin pH indicators [40]. Multiple forces continuously shape packaging evolution: scientific breakthroughs creating new functional horizons; regulatory pressures dictating acceptable safety margins; cultural values prioritizing freshness transparency; and environmental realities, steering production away from persistent synthetic waste streams. This cumulative pathway reveals that current smart packaging sophistication did not emerge abruptly but was rooted in centuries-long adaptation cycles responding to changing material science capabilities and societal priorities. Tracing this lineage also underscores an underlying continuity: whether through clay jars safeguarding wine millennia ago or present-day nanocomposite pouches tracking microbial activity via sensor arrays, the central aim persists, protecting food’s integrity until consumption. The technical toolkit has vastly expanded over time, but its historical anchors remain connected through shared objectives shaped by necessity at each stage of advancement.

### 2.2. Transition from Passive to Active Packaging

**Figure 2:**
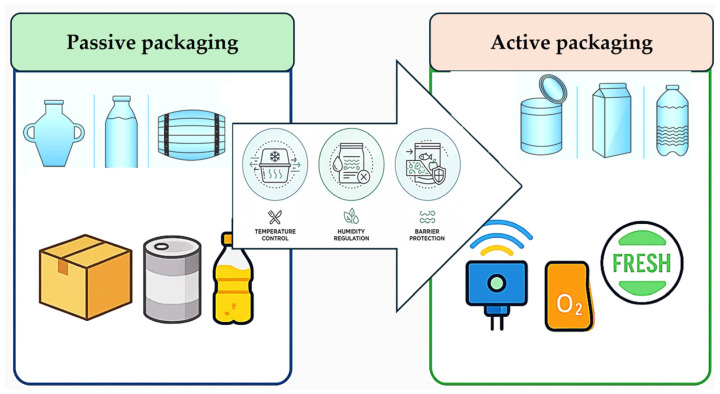
Transition from passive to active packaging.

The shift from passive packaging formats, characterized by their static and inert roles, to active systems capable of modulating a product’s microenvironment appears closely tied to technological progress and evolving consumer and regulatory pressures. As noted in Section 2.1, traditional packaging primarily functions as a physical safeguard, providing structural containment and protection from external contamination [8,32,41]. Passive modes were effective for basic preservation but lacked adaptability to changing conditions during distribution or storage. They relied on initial sealing processes without later interaction, once packaged, the internal atmosphere or composition of the food remained unchanged until opening. Active packaging emerged as a response to limitations inherent in purely passive approaches (Figure 2).

Typical innovations include oxygen scavengers, moisture absorbers, antimicrobial agents, ethylene removers, and controlled-release preservatives. Technological designs range from simple sachets containing desiccants that regulate humidity to complex multilayer films engineered with embedded functional biomolecules [10,29,42]. Within this category, the incorporation of compounds such as antimicrobials and antioxidants directly into polymer matrices exemplifies how materials science has enabled active functionalities beyond traditional passive roles. The primary catalyst for this shift is the recognized link between packaging performance and food-safety requirements. Foodborne pathogens and spoilage organisms can proliferate when environmental conditions within a package become favorable [17,30,43]. Active technologies mitigate this risk by controlling factors such as humidity or gaseous composition. Moisture regulation, for example, can involve hydrophilic salts dispersed within foamed polymer layers produced via thermoforming; these alter water vapor transmission rates while matching physical resilience requirements [27,32]. Such interventions not only slow microbial proliferation but also maintain desirable texture and sensory attributes. Cost considerations have historically limited active components to high-value or sensitive products. However, advances in polymer processing, such as hot melt extrusion for uniform dispersion of active fillers, are making these technologies more adaptable across product categories. Integration challenges remain, particularly regarding migration risks for bioactive agents into the consumable portion of food [44]. Regulatory bodies closely evaluate these risks as they are directly aligned with public health policy. This transition also reflects increasing sophistication in the understanding of packaging–food–environment interactions. Active systems function within dynamic equilibrium frameworks, requiring the packaging to respond to changes arising from internal biological activity (e.g., respiration in fresh produce) or external perturbations, such as fluctuations in ambient temperature [41]. Modified atmosphere packaging (MAP), often paired with oxygen scavengers, represents a hybridized evolution toward active control mechanisms intended to sustain optimal gas balances inside sealed units [45]. Consumers’ demand for fresher products over extended timelines has heightened industry interest in visible quality assurance markers embedded within packages. Though intelligent indicators are distinct from purely active agents, combining them with interactive preservation functions amplifies perceived value. For instance, colorimetric freshness sensors embedded into edible biopolymer films not only monitor degradation markers but also coexist with antimicrobial additives targeting spoilage at the source [19,46]. This synthesis blurs strict categorical boundaries between “active” and “intelligent,” forging hybrid packages tailored toward multi-level quality maintenance strategies. Studies record consumer concerns regarding reliability and universality, for example, doubts over whether oxygen-scavenging sachets perform equally well for diverse foods differing in moisture content or density [5,47,48]. Looking deeper at industrial implications, transitioning into active formats forces a reevaluation of supply chain logistics. Since active systems continue to alter conditions within packages post-sealing, storage duration predictions must incorporate kinetic models describing agent depletion rates or diffusion coefficients through various layers. Offering clear labeling on active functioning may encourage adoption while mitigating suspicion regarding unseen chemical processes occurring during storage. The shift toward active packaging configurations is driven largely by environmental concerns related to food waste reduction and safety enhancement, making a return to purely passive designs unlikely. Transforming conventional passive systems into more adaptive solutions offers several tangible advantages, including reduced spoilage-related losses, extended shipping distances, decreased reliance on synthetic preservatives, and improved sensory quality upon opening [2,9,24,35]. Still, balancing regulatory compliance on migration limits with technological efficiency remains critical to sustaining widespread application growth. Overall, the movement away from inert packaging characterizes an inflection point where material sciences intersect operational realities under increasingly stringent sustainability mandates. This point toward a future dominated by integrated systems combining preservation chemistry, responsive mechanics, and informational signaling, a continuum evolving well beyond conventional cradle-to-consumption philosophies inherited from earlier passive paradigms.

## 3. Classification of Packaging Systems

The evolution of technologies applied to food packaging has culminated in the development of smart packaging, a concept that integrates monitoring, communication, and product-condition-responsive functionalities. In this context, examining current smart packaging applications in the food industry becomes essential for understanding how these systems contribute to enhanced safety, extended shelf life, and improved traceability (Table 1). Furthermore, a systematic classification of the various types of smart packaging, structured according to their functional principles and operational performance (Table 2), enables a comparative assessment of existing solutions and supports the identification of future development directions in the field.

Cost and limitations

Passive packaging: Generally low to moderate cost; materials already industrially adopted, minimal cost increase compared to conventional packaging.

Active packaging: Higher material cost (active agents/antimicrobials); regulatory approval and migration studies may limit scalability and global adoption.

Intelligent packaging: Indicators increase unit costs; stability, sensitivity, and reliability under refrigerated conditions remain limitations [7,10,16,58].

Hybrid Smart Systems: Higher implementation costs due to sensors, data infrastructure and RFID systems; limited by long-term sensor stability, and cold-chain variability.

### 3.1. Passive Packaging Systems

Passive packaging systems represent the earliest form of food packaging, designed primarily as inert barriers protecting contents from harmful external conditions. In these systems, materials are chosen for their mechanical strength, barrier efficiency, and durability, not for interactive functions. Once sealed, the internal state of the product remains largely stable until consumption [41,88].

Their protective role focuses on containment, shape retention, and prevention of contamination, while minimizing exposure to moisture and gases through impermeable layers. Performance depends on intrinsic material properties such as tensile strength, oxygen transmission rate (OTR), water vapor transmission rate (WVTR), and UV resistance. Common materials include glass, metal cans, paperboard laminates, and polymers like PET, HDPE, PP, and LDPE, valued for their low cost and manufacturing versatility [51,79,89].

However, their inert nature affords only static protection, meaning that temperature or humidity fluctuations can still accelerate food degradation [36]. Passive systems remain suitable for stable products, such as dry goods or carbonated beverages, where shelf-life expectations align with controlled storage and distribution conditions. Yet when handling practices or temperature levels deviate from these conditions, such packaging lacks the capacity to adapt and mitigate spoilage.

Unlike active or intelligent packaging, passive systems lack direct freshness or safety indicators; consumers rely solely on expiry dates, which may not reflect real-time product quality. In the context of environmental sustainability, these systems face scrutiny for their reliance on non-degradable petroleum-based materials, which persist for decades after disposal. Advances in biopolymers and nanocomposite reinforcements, using additives such as nanoclays or cellulose nanofibrils, show promise in improving biodegradability and mechanical strength [29,45,90,91].

Despite these limitations, passive packaging retains advantages: simplicity, reliability, and low regulatory complexity as it does not involve the intentional migration of active substances. These properties make it suitable for long-term storage, such as military rations or humanitarian aid, where robustness outweighs innovation [92].

From a design perspective, optimizing passive packaging involves balancing barrier trade-offs, for instance, low WVTR for hygroscopic products versus high OTR control for oxygen-sensitive foods. Aluminum laminates enhance gas impermeability but complicate recyclability [42].

Recent developments have explored hybrid approaches, combining inert containers with external monitoring tags or removable active inserts [9,51,93,94]. Though gradually replaced in high-tech sectors, passive packaging remains fundamental to global food distribution for its proven reliability, economic efficiency, and role as a baseline for evaluating newer technologies.

### 3.2. Active Packaging Systems

Active packaging systems differ from the inert barriers described in Section 3.1 by introducing controlled interactions between the package, the product, and its microenvironment. Their goal is to extend shelf life, improve sensory quality, and enhance food safety. These systems incorporate biological, chemical, or physical agents into the packaging structure to regulate internal conditions over time. This transforms packaging from a passive container into an active preservation tool.

The main functional categories include scavenging, releasing, blocking, and regulating systems.

Active packaging systems employ a range of functional mechanisms designed to control the internal environment of food products. Scavenging technologies remove undesirable substances such as oxygen, moisture, carbon dioxide, ethylene, and various volatile compounds. Oxygen scavengers, commonly based on iron or titanium dioxide reactions, slow oxidative deterioration and microbial proliferation. Moisture absorbers, including desiccant sachets or hygroscopic polymer layers, prevent textural defects such as staling in crispy products or caking in powdered foods.

In addition to scavenging, releasing systems deliver beneficial compounds into the food matrix or headspace. Antimicrobial release agents such as silver nanoparticles, organic acids, nisin, and zinc oxide have demonstrated effectiveness in suppressing bacterial and fungal growth. Similarly, CO_2_ emitters formulated from citric acid and sodium bicarbonate are widely used in modified-atmosphere packaging (MAP) for fish and poultry, where controlled gas evolution helps maintain product freshness.

Other approaches focus on blocking or regulating key degradation pathways. Ethylene blockers delay fruit ripening, while antioxidant films limit lipid oxidation in high-fat foods. Regulating systems further contribute to product stability by maintaining consistent humidity or gas levels, thereby reducing mold development and quality loss [14,29,45,50,55,95,96,97]. Active agents are commonly incorporated into polymer matrices through extrusion or coating techniques, enabling controlled antimicrobial or antioxidant functionality. For example, lysozyme-loaded PVA films exhibit targeted antibacterial effects, whereas chitosan–lactoperoxidase composites demonstrate antifungal efficacy in fruit preservation [98]. Although these systems show clear performance advantages, a major industrial challenge remains achieving uniform dispersion of active compounds, as heterogeneity can lead to inconsistent release rates and variable microbial suppression. This limitation is repeatedly highlighted across studies and represents a key barrier to large-scale implementation.

Nanotechnology further enhances the efficiency of active systems by increasing surface reactivity and reducing the required dosage of functional compounds. Nanocomposites incorporating chitosan, nanoclays, or metal-oxide nanoparticles consistently improve barrier properties and antimicrobial potency [99]. These hybrid materials introduce multifunctionality, simultaneously modulating gas permeability, moisture transfer, and microbial inhibition, yet also raise concerns regarding cost, regulatory approval, and potential nanoparticle migration, underscoring the need for harmonized safety frameworks.

Recent developments combine active and intelligent features, reflecting a shift toward integrated preservation-monitoring platforms. For instance, modified-atmosphere packaging (MAP) can incorporate CO_2_ emitters alongside colorimetric indicators to detect gas leakage or product aging. While such systems offer substantial advantages in reducing spoilage, they require food-specific optimization: oxygen scavenger capacity differs markedly between high-fat products and fresh produce, and inappropriate calibration may result in under- or over-performance. Kinetic modeling based on Fickian diffusion principles supports this tailoring by predicting release behavior under different temperature, humidity, and permeability conditions [31,46,100].

Sustainability considerations increasingly influence material selection, pushing research toward biodegradable matrices compatible with active components. PLA- and starch-based polymers represent promising candidates; however, their integration with reactive agents remains hindered by issues of compatibility, mechanical stability, and moisture sensitivity [101]. Hybrid systems such as alginate-based carriers combined with natural antimicrobials offer a pathway toward compostable yet functional packaging. Still, ensuring biodegradability without compromising controlled-release efficiency remains an unresolved challenge and a central direction for future research.

Commercial adoption is constrained by cost, processing complexity, and consumer perception. Advanced techniques such as co-extrusion and microencapsulation significantly elevate production expenses, limiting their feasibility to high-value perishable foods such as meats and fruits [102]. In parallel, public acceptance depends on effective communication addressing concerns related to perceived “chemical additives” in direct contact with food [38,44,103]. Comprehensive risk assessment—through migration testing, accelerated aging, and field trials monitoring microbial reduction and active-agent depletion—remains essential to building trust and regulatory compliance. Current legislation requires active components to remain within strict migration limits; however, divergent national frameworks hinder international commercialization, reinforcing the need for harmonized global standards.

As a multidisciplinary innovation, active packaging integrates chemistry, materials science, and microbiology to create systems capable of responding to environmental fluctuations. Beyond extending shelf life and reducing food waste, these responsive technologies support market expansion for perishable products and align global sustainability goals. Nonetheless, balancing performance, safety, cost, and biodegradability remains the key research frontier that will determine whether active packaging can serve as a viable alternative to conventional plastics.

### 3.3. Intelligent Packaging Systems

**Figure 3:**
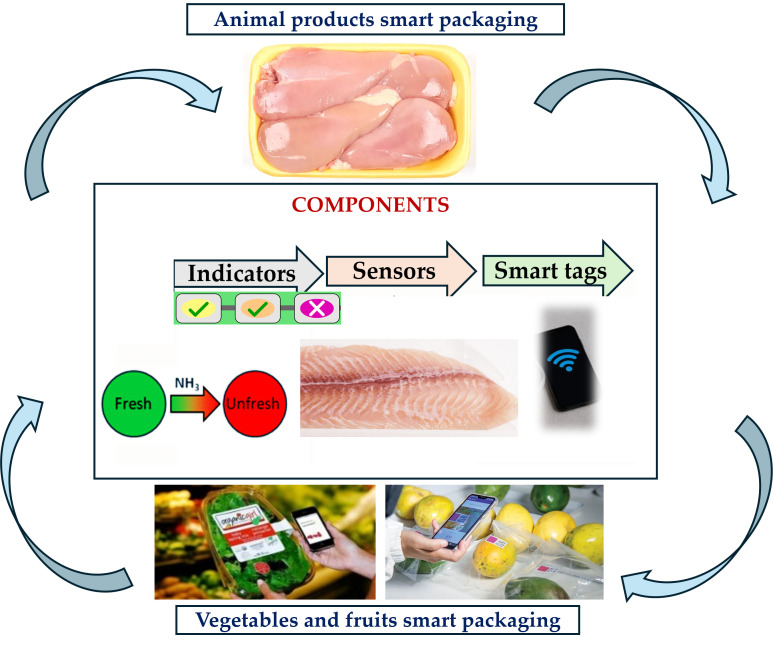
Intelligent packaging systems.

Intelligent packaging systems extend the concept of active preservation by embedding mechanisms that can monitor, record, and communicate information regarding the product’s status or the conditions surrounding it (Figure 3). Unlike purely active designs, their primary function is informational: enabling stakeholders across the supply chain to respond to quality changes in real time rather than passively relying on predefined shelf-life estimates.

This capacity is crucial in modern logistics, where unpredictable environmental fluctuations during transport can drastically influence product integrity. The technological backbone of intelligent packaging comprises three categories: indicators, sensors, and data carriers [27,37,93,104]. Indicators provide immediate, usually visual feedback about specific attributes, for example, color shifts linked to cumulative temperature exposure in time–temperature indicators (TTIs), or changes triggered by volatile compounds signaling microbial spoilage. Sensors go beyond simple indicators by detecting a wider range of parameters such as temperature profiles, relative humidity levels, oxygen concentration, carbon dioxide composition, or even pH shifts within the package environment [36,105]. Data carriers encompass tools such as RFID tags and barcodes designed to store and transmit product-related data through various stages of logistics. A key example is the TTI predictor technology, which determines dynamic shelf lives based on initial reference temperature values. Integrating spectrophotometric readings from indicator labels at each stage of distribution enables real-time adjustments to the remaining shelf life. This granular monitoring enables decision-makers to redirect products close to expiring towards nearby markets or launch rapid sales promotions [96,106]. TTIs essentially link laboratory models with field observations through straightforward visual indicators or digital integration. Recent advances combine intelligent indicators with active preservation methods, forming hybrid smart packaging capable of simultaneously extending freshness and reporting degradation. For instance, gas concentration indicators embedded into fresh meat packaging may shift color when microbial respiration increases CO_2_ above safe thresholds; concurrently, antimicrobial films can suppress spoilage rates until intervention occurs. These designs rely heavily on synchronization between signal output and actual sensory deterioration so that the information presented aligns closely with food safety reality [29,60,107]. The application spectrum has greatly expanded due to commercial innovations such as Turbotag, an RFID tag that monitors temperature and logs a commodity’s thermal history for visibility across the entire supply chain. Some RFID-enabled sensors, beyond just monitoring capabilities, can identify pathogenic microbes by attaching them to artificially created virus structures within sealed detection areas. The microbial event triggers generate electronic signals that can be detected by portable systems at inspection stations, especially for high-risk foods like ready-to-eat meats or seafoods, where contamination can have severe consequences [108]. Intelligent packaging systems also employ bio-based sensor paradigms using natural polymers and pigments responsive to biological changes. For example, freshness indicators leveraging pH-sensitive anthocyanins change hue upon detecting acids produced during protein decomposition in fish storage environments [30,100]. Bio-based sensors aim not only to provide accurate detection but also to align with sustainability requirements through compostable substrates and minimal chemical residues post-disposal. While such approaches appear promising environmentally, stability under fluctuating temperatures or light exposure remains under study and could limit broader market rollout without enhancement. The advantages attributed to intelligent packaging include heightened transparency in product quality verification across diverse handling contexts and extended ability to mitigate food waste through proactive responses. Supply chain actors gain early alerts regarding deviation from optimal ranges, preventing spoiled goods from reaching consumers, and enhancing compliance with regulatory requirements for cold chain documentation during audits. However, these strengths are tempered by disadvantages such as a narrow focus on certain devices (e.g., solely tracking thermal events without addressing non-temperature-related degradation) and increased unit cost compared to conventional static formats [94,109]. Implementation of industry-wide technologies can be hindered when the costs associated with setup and integration far outweigh perceived advantages among parties without experience with the expected returns on investment. A persistent challenge is standardization: the lack of uniform guidelines makes it difficult to choose among diverse technologies that offer similar results but have different operational parameters or communication protocols. Ultimately, intelligent packaging situates itself as both a diagnostic tool and facilitator for adaptive logistics strategies, linking sensory science with digital traceability architectures capable of responding within hours rather than days to shifts in product condition during transit or storage.

## 4. Materials and Technologies for Smart Packaging

The materials and technologies for smart packaging encompass innovative biodegradable substrates, active components, and advanced sensing systems that enhance food preservation, safety, and real-time interaction throughout the supply chain.

### 4.1. Innovative Packaging Materials

Innovative packaging materials integrate biodegradable polymers, nano-enhanced structures, and environmentally friendly composites designed to improve product protection while supporting sustainability and reducing ecological impact.

#### 4.1.1. Biodegradable and Sustainable Materials

Biodegradable and sustainable materials have emerged as a critical focus in packaging innovation. The move away from petroleum-derived polymers stems from concerns about their persistence in natural ecosystems and the resultant pollution impact. Biodegradable alternatives, particularly those derived from renewable sources, aim to balance mechanical performance, barrier properties, and processability with compostability or degradability after disposal. This shift implies a rethinking of material selection criteria, where the end-of-life environmental impact becomes as important as the in-use functionality. A notable category within this area is starch-based bioplastics, such as those produced from corn starch waste or direct extraction from corn plants. These exhibit very high biodegradability profiles while using agricultural by-products that might otherwise contribute to waste streams [15,34,110]. Corn starch packaging can be formed into containers or films using heat and is suitable for use with food. Their stability under humid storage conditions is limited by intrinsic hydrophilicity, so they often need to be blended with other biodegradable polymers or undergo surface modifications to prevent moisture uptake without sacrificing compostable properties [101,111,112]. Similarly, polybutylene succinate (PBS) is an example of a synthetic but biodegradable polyester developed for packaging applications. PBS resists heat and light exposure and maintains favorable processability characteristics in extrusion or film blowing [29]. Nevertheless, its lower impact strength and tear resistance constrain application scope unless reinforced through copolymerization or inorganic filler addition. Highly amorphous polyvinyl alcohol (commonly referred to as polymer-G) offers another candidate, strongly biodegradable yet mechanically flexible, but must reconcile solubility traits with water resistance when employed for moist products [113]. From an ecological standpoint, plant-material-based packaging offers dual benefits: it leverages naturally occurring celluloses, hemicelluloses, or lignins to fabricate structures that degrade within environmental cycles while contributing minimal greenhouse gas emissions during production. When cultivated under sustainable agricultural practices, such feedstocks avoid competition with essential food supply resources [114,115]. The use of 3D printing technologies on plant-based filaments provides precision control over material use efficiency, reducing manufacturing scrap rates while enabling bespoke package geometry adapted to product-specific shapes. Advances in nanotechnology have further enhanced the viability of biodegradable materials by augmenting barrier functions without resorting to persistent synthetic layers [116]. Adding nanoparticle reinforcements like nanoclays to starch or PLA matrices enhances tensile strength and reduces gas transmission rates, characteristics that are typically difficult to obtain in fully biodegradable systems. These improvements expand their capabilities into areas that need stricter preservation standards, such as fresh produce or high-fat products susceptible to oxidative rancidity [94,117]. Conserving resources and reducing energy intensity can be achieved by utilizing recycled organic material streams, such as a blend of reclaimed paper pulp and natural fiber composites, instead of extracting virgin materials. Consumer perception plays an equally strong role; awareness campaigns highlighting successful examples of biodegradable packaging in retail environments could enhance receptivity while mitigating skepticism related to durability or product safety. Government legislation exerts significant influence over adoption trajectories. Regulatory incentives such as banning single-use plastic bags or imposing plastic taxes encourage industry investment in eco-packaging solutions [112,118]. Companies that switch to biodegradable products can meet increasing consumer demands for environmentally responsible practices and comply with changing waste disposal regulations. Not all biodegradable materials perform equally well across a range of packaging situations. Biopolymers sensitive to moisture may perform well in dry goods packaging but struggle when sealing perishable goods that are transported in humid environments [30,34]. Including antioxidant releasers within bio-film matrices extends shelf-life without needing non-degradable carriers; this can be achieved by encapsulating green tea extract into multilayer PLA films for oxidative stability control. Similarly, antimicrobial peptides embedded inside edible alginate coatings provide both bio-protection and full environmental reconciliation upon disposal [94,119,120]. These integrations allow smart packaging paradigms to operate within fully green material footprints rather than conventional petrochemical baselines. There remains an open scientific challenge concerning the unintended impacts of biodegradable systems throughout their full lifecycle. Factors such as methane generation during anaerobic degradation or incomplete breakdown under suboptimal conditions warrant closer examination lest environmental gains be offset by side effects elsewhere in waste chains [114]. Laboratory simulations coupled with field-scale validation could clarify optimal contexts ensuring maximal emission reductions alongside safe reabsorption into natural nutrient cycles. The broader path indicates convergence between performance requirements characteristic of conventional plastics and degradability requirements influencing the development of future regulatory frameworks. Research collaborations involving polymer scientists, logistics providers, policymakers, and consumer advocacy groups appear likely to speed up the refinement process required for the large-scale rollout beyond environmentally aware markets.

#### 4.1.2. Emerging Nanomaterials: Metal–Organic Frameworks (MOFs) and Quantum Materials for Smart Packaging

**Figure 4:**
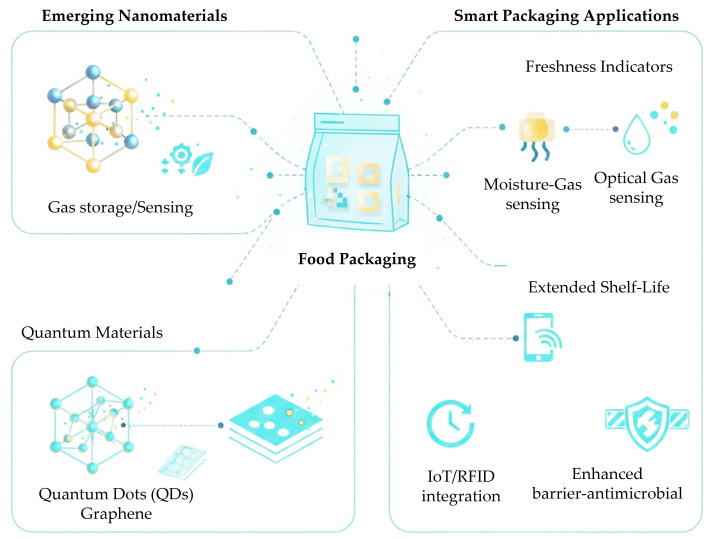
Innovation in smart food packaging.

Emerging nanomaterials such as Metal–Organic Frameworks (MOFs) and quantum-derived nanostructures offer a viable pathway toward replacing conventional plastic packaging by enabling biodegradable, high-performance, and multifunctional smart packaging systems that preserve food quality and extend shelf life. Also, nanomaterials have emerged as a transformative element in packaging innovation, providing functionalities that surpass conventional materials and complementing biodegradable strategies (Figure 4).

Emerging nanomaterials for smart food packaging can be grouped into three main categories: (i) Metal–Organic Frameworks (MOFs), which function as gas-scavenging and antimicrobial platforms due to their high specific surface area and tunable porosity; (ii) quantum-derived nanomaterials, such as carbon dots and graphene quantum dots, mainly providing antimicrobial, antioxidant and UV-blocking performance; and (iii) hybrid MOF–polymer or MOF–metal oxide systems that combine multiple active and intelligent functionalities within bio-based matrices. Among these classes, MOFs represent crystalline porous networks generated through the coordination of metal ions with organic ligands, resulting in structures with an adjustable pore size and surface chemistry. Such tunability enables MOFs to operate as oxygen, moisture and ethylene scavengers, or as carriers for antimicrobial and antioxidant agents, thereby supporting both active and intelligent functions in food packaging applications. Recent reviews have emphasized the potential of MOF-based systems to operate as antimicrobial agents, oxygen and ethylene scavengers, and moisture absorbers in active food packaging applications [86,121].

MOF-based active-packaging materials have been explored for post-harvest fruit and vegetable preservation. Fu et al. (2023) summarized MOF-loaded films and coatings designed to adsorb ethylene and delay ripening, showing that properly engineered MOFs can significantly extend the post-harvest shelf-life and maintain the quality attributes of fresh produce [122]. More recent studies have focused on biopolymer matrices incorporating MOF-based nanofillers, for example, cellulose nanofiber/gelatin films containing bismuth MOF–TiO_2_ hybrids that combine ethylene scavenging, photocatalytic activity and antimicrobial protection, thus slowing fruit ripening while limiting microbial spoilage [50,72,112,123].

Other studies have demonstrated cerium-based MOFs embedded in food-contact films as efficient ethylene scavengers with added antioxidant and antibacterial performance, further illustrating the versatility of MOFs as multifunctional nanofillers for smart packaging systems [124].

In parallel, quantum-derived nanomaterials, particularly carbon dots (CDs), carbon quantum dots (CQDs) and graphene quantum dots (GQDs) emerge as promising, cost-effective alternatives or complements to MOFs in active and intelligent packaging. Carbon dots are ultra-small carbonaceous nanoparticles with abundant surface functional groups, excellent aqueous dispersibility, strong and tunable photoluminescence, and generally low toxicity and good biocompatibility [125].

When incorporated into biopolymer films, CDs can endow the packaging with multiple functionalities, including UV-blocking, radical scavenging (antioxidant activity) and broad-spectrum antimicrobial effects, while at the same time improving the mechanical strength and barrier properties [34,126]. Recent reviews have highlighted that CD-based systems can be used not only as active agents in films and coatings, but also as sensing elements for intelligent packaging and food quality monitoring [127].

Several recent experimental studies demonstrate the practical feasibility of quantum-derived nanomaterials in food packaging. Priyadarshi et al. (2024) reported edible coatings containing carbon quantum dots applied directly on fresh and fresh-cut fruits, showing that CQDs can be safely used in contact with food and that their antibacterial and antioxidant activities effectively delay spoilage and preserve quality [128].

Singh et al. (2024) developed gelatin-based films enriched with green carbon quantum dots, which significantly slow the decay of fresh-cut apples in refrigerated storage and improve the antioxidant capacity of the films [129]. According to Riahi et al. (2025), red pepper waste-derived carbon dots were successfully incorporated into sodium alginate/gelatin matrices to develop multifunctional active packaging films with enhanced antimicrobial, antioxidant, and UV-barrier performance, demonstrating that biowaste-based nanomaterials can significantly extend fruit shelf life while contributing to sustainable food waste management [130,131].

Graphene quantum dots have also been introduced into biodegradable packaging matrices, where their ability to generate reactive oxygen species and interact with microbial membranes leads to strong antibacterial and antifungal activity, together with UV shielding and improved oxidative stability of the packaged food [132].

MOFs and quantum-derived nanomaterials comprise two distinct classes of emerging nanofillers for smart food packaging. MOFs offer highly customizable porosity and sorption properties, making them suitable for use as gas scavengers and controlled-release systems for active compounds, whereas carbon-based quantum materials exhibit robust optical responses, effective UV blocking capabilities, and antioxidant and antimicrobial effects, and can frequently be synthesized from low-cost biowaste precursors [132,133,134].

By integrating these advanced nanomaterials into biodegradable or bio-based polymer matrices, recent research demonstrates that it is possible to design multifunctional packaging films and coatings that simultaneously enhance food safety, extend shelf-life and contribute to more sustainable and efficient packaging solutions.

### 4.2. Embedded Sensing Technologies

Embedded sensing technologies integrate chemical, physical, and biological sensors into packaging systems, enabling continuous monitoring of food quality, environmental conditions, and potential contamination in real time.

#### 4.2.1. Chemical Sensors for Food Quality

Chemical sensors for food quality operate at the intersection of analytical chemistry, material science, and food safety engineering, using selective detection mechanisms to identify or quantify compounds that signal deterioration, contamination, or suboptimal storage conditions. They transform chemical interactions in the packaging environment into measurable outputs, often electrical or optical, that can be processed and interpreted by end users or automated systems. This capability strengthens the functionality of smart packaging by enabling proactive interventions when quality degradation begins, rather than relying on fixed-expiry assumptions. Within packaged food environments, certain gases and volatile compounds act as reliable indicators of microbial growth or chemical changes. Carbon dioxide (CO_2_) is widely monitored because its accumulation inside sealed packages often results from respiration in fresh produce or metabolic activity by spoilage microorganisms in protein-rich products. Non-dispersive infrared (NDIR) sensors detect CO_2_ through spectroscopic absorption at specific wavelengths, converting changes in transmitted light intensity into concentration readings [29,107,135]. These sensors are well-suited for integration into rigid or flexible packaging formats given their non-contact nature, though calibration stability under temperature fluctuations must be carefully managed to avoid false readings. Optical oxygen sensors are another strand of chemical sensing techniques that contribute to food quality assessment. They typically rely on absorbance changes or luminescence quenching when sensor dyes interact directly with O_2_ within the headspace of a package [31,136,137]. Since oxygen levels strongly influence oxidative rancidity, color stability, and aerobic microbial proliferation, monitoring them allows producers to confirm that modified atmosphere packaging (MAP) conditions remain intact throughout distribution. In perishable goods, such as vacuum-packed cured meats, where even trace oxygen can shorten shelf life, these sensors serve both as a diagnostic and compliance verification tool against mishandling or seal failure [41,138]. Functional diversification has extended chemical sensors toward detecting ethylene gas emitted by certain fruits during ripening. Ethylene accelerates senescence processes; its timely removal or suppression can extend fresh windows for retail display [114,139]. Sensor arrays identifying ethylene concentrations facilitate coordinated operation with active components, ethylene absorbers, or blockers to maintain optimal ripening stages until sale. Analogously, volatile amines, such as trimethylamine and ammonia arise during fish spoilage; pH-sensitive dyes embedded into polymer films or labels can respond to their presence through distinct color shifts [100,114]. This approach offers direct freshness cues without the need for specialized readers, enhancing transparency for consumers inspecting products before purchase. Sensor elements are frequently combined with conductive polymers, piezoelectric crystals, or metal oxide semiconductor field-effect transistors to produce amplified electronic signals because of analyte interaction. These systems can take advantage of selective chemistry at the nanomaterial level, such as functionalized graphene sheets engineered to absorb target molecules while changing electrical resistance in proportion to concentration [140,141]. The use of carbon nanomaterials is particularly attractive owing to their high sensitivity and mechanical compatibility with thin film substrates. While many chemical sensors focus on single-analyte detection for specific reasons, multisensory arrays capable of simultaneous monitoring across a suite of volatile markers emerging as powerful tools for complex food matrices. By constructing data fingerprints based on proportional responses among multiple detection channels, often referred to as “electronic noses”, packaging systems can differentiate between similar spoilage pathways caused by different microbial communities or physical stresses. The application of machine learning algorithms applied to these sensor datasets improves the predictive accuracy of the remaining shelf life over traditional threshold-based approaches. From a manufacturing perspective, embedding chemical sensors directly within package walls avoids additional insertion steps but requires careful consideration of sensor stability during forming or sealing processes [42,142]. Extrusion-based incorporation requires thermally stable sensing chemistries; conversely, post-production coating methods permit heat-sensitive reagents but may limit adhesion durability under handling stresses. Packaging transparency also influences sensor choice: optical detection systems relying on visible cue presentation must position sensing layers in a manner that does not obscure them by opaque design elements. The adoption of biodegradable substrates combined with non-toxic sensing agents aligns output devices with circular economic objectives without abandoning functional integrity over service life. Bio-based pigments responsive to pH changes during spoilage leverage natural colorants such as anthocyanins, safe within dietary contexts, while avoiding synthetic dye residues post-disposal [30]. Emerging research suggests pairing nanoscale sensing elements directly with wireless data transmitters such as RFID chips embedded in smart packaging ecosystems. This configuration transforms static measurements into continuous condition streams that are remotely accessible along the logistics chain [33,37]. Such connectivity supports rapid response actions such as rerouting shipments when cumulative spoilage indicators breach defined thresholds, mitigating waste before products reach retail shelves. As smart packaging continues to expand toward multifunctional platforms that combine preservation chemistry with real-time diagnostics, chemical sensors will likely evolve into integrated microsystems that offer precise environmental mapping inside each packaged food unit [17,21,143,144]. This evolution opens pathways not only for enhanced safety assurance but also for optimized resource management across globalized distribution networks where minute deviations during storage could otherwise propagate substantial loss events if undetected until the terminal inspection stages.

Costs and Limitations

However, the large-scale adoption of chemical sensing technologies in food packaging remains limited by the high unit cost of sensor components, the need for specialized printing or integration processes, and additional IoT infrastructure for data transmission and management. Moreover, current chemical sensors may face challenges related to long-term stability, calibration during storage, and reliable performance under variable temperature and humidity conditions, which further constrain widespread commercial implementation [7,10,16,58].

#### 4.2.2. Temperature and Humidity Sensors

Temperature and humidity sensors embedded in food packaging play a key role in monitoring environmental conditions that affect food safety, quality, and shelf life. Their integration transforms packaging into a dynamic feedback system that alerts users to temperature or moisture deviations during storage and transport.

Temperature fluctuations are a major cause of food deterioration. High temperatures promote microbial growth, lipid oxidation, and texture loss, whereas poor cold chain control can cause thaw–refreeze cycles that damage the structure. Time–temperature indicators (TTIs) work on the principle that heat exposure over time reflects product degradation. Polymer-based TTIs use dyes that migrate or change color according to thermal history. More advanced types use enzymatic reactions whose rates vary with temperature, enabling the estimation of shelf life [4,14,36,62].

Electronic temperature sensors provide higher accuracy. Miniature thermistors or resistance detectors (RTDs) on flexible circuits can record continuous data and transmit it wirelessly through RFID modules. These systems verify cold-chain compliance for perishables such as seafood and dairy, sending alerts when limits are exceeded [31,145]. Predictive algorithms support logistics decisions, such as rerouting or prioritizing products nearing degradation.

Humidity also affects food quality. High humidity encourages mold growth and clumping, whereas low humidity causes drying and hardening. Humidity sensors detect these variations through capacitive sensors (measure changes in the dielectric constant as water is absorbed); resistive sensors (detect conductivity shifts in hydrophilic polymers); optical hygrometers (track light scattering from condensation) [58,60,65].

Flexible packaging can be integrated with thin-film capacitive RH sensors without altering its structure. Combining them with desiccant components generates self-regulating microenvironments. A film that incorporates silica gel pockets can initiate additional drying agents when humidity surpasses specified limits, thus maintaining the crispness of snacks throughout the distribution process [130,146].

Maintaining sensor calibration is crucial. Temperature sensors must remain accurate across −20 °C to +40 °C, while humidity sensors must minimize hysteresis—drift caused by repeated wet–dry cycles. Another challenge is the power supply: passive RFID sensors harvest energy from readers but need proximity, whereas printed thin-film batteries or solar cells offer independence at higher cost [106].

When integrated into intelligent packaging, temperature and humidity data become more valuable. Correlating humidity spikes with CO_2_ readings, for instance, can reveal spoilage caused by microbial activity [95,99,147]. Multi-sensor systems enhance predictive quality control and reduce false alarms.

In modified atmosphere packaging (MAP), these sensors confirm the gas balance and detect leaks or unwanted permeability. Reliable performance requires material compatibility, flexible circuits must endure sealing temperatures, and food-safe sensor coatings [45].

Market-ready examples include luminescent ZnO nanoparticle films embedded in polymers, which change brightness with temperature, and color-shifting nanomaterials that indicate humidity levels in bulk goods [95,119,148]. Such low-cost visual indicators allow easy quality checks during transport.

Future designs will likely feature printed biodegradable sensor arrays that combine temperature, humidity, pH, and gas detection. These smart sustainable systems align with the circular economic goals. Correctly implemented, temperature and humidity sensing strengthens proactive quality assurance from production to retail, reducing waste and enhancing consumer trust.

Costs and Limitations

Temperature and humidity sensors currently add a noticeable cost to smart food packaging, with low-cost printed temperature indicators ranging from a few cents per unit while electronic temperature–humidity modules or RFID-based loggers involve higher costs, depending on functionality and volume.

Passive RFID sensors are battery-free, low-cost and can be embedded in food packaging. An important direction in developing such sensors is to find a biocompatible, food-safe, and temperature-sensitive smart material. However, limitations such as calibration drift, sensitivity to variable storage conditions, energy demand for continuous monitoring, and long-term signal stability continue to restrict large-scale commercial adoption in conventional food supply chains.

### 4.3. Benchmarks and Comparative Evaluation of Smart Food Packaging Systems

To contextualize recent advances in smart food packaging, it is important to identify relevant benchmarks and compare their characteristics with those of emerging nanomaterial-based systems. Conventional passive packaging based on plastics, paper, glass, or metal still represents the dominant benchmark in the food industry, providing basic mechanical strength and barrier properties but offering limited active functionality and no real-time quality monitoring [149].

Ahmed et al. (2022) systematically reviewed active packaging systems for food quality and safety, showing that incorporating oxygen scavengers, antimicrobial agents, and antioxidant components into packaging films can substantially reduce microbial growth and oxidative deterioration compared with conventional packaging, leading to significant shelf-life extension across diverse food categories [150].

Mkhari et al. (2025) highlighted a wide range of intelligent packaging solutions, including colorimetric freshness indicators, gas sensors, and RFID-based systems that allow real-time monitoring of food quality and storage conditions, offering a distinct benchmark for comparison with other developed smart packaging technologies [151]. Environmental impact assessments, such as the comparative study by Srivastava et al. (2024), have shown that active biocomposite packaging can reduce environmental burdens relative to conventional plastic packaging, while maintaining or improving preservation performance [152].

Table 3 provides a systematic overview of the identified benchmark packaging system types (passive, active, and sustainable) and delineates their characteristic features relative to Smart Food Packaging. Furthermore, the analysis explicitly outlines indicative cost considerations associated with the material composition and technological complexity inherent to each system’s application and manufacturing process.

Ultimately, while smart packaging offers a revolutionary leap in real-time safety assurance and supply chain transparency, its commercial viability and widespread adoption are presently constrained by its significantly higher unit cost and inherent complexities in achieving environmental compliance compared to established Passive and Active packaging benchmarks.

## 5. Applications of Smart Packaging in the Food Industry

Smart packaging applications in the food industry span a wide range of functions, from extending shelf life and ensuring product safety to enhancing traceability, transparency, and consumer interaction through advanced digital and sensing technologies.

### 5.1. Shelf-Life Extension

**Figure 5:**
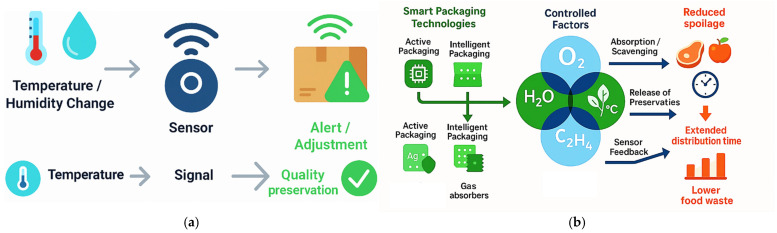
Packaging methods for the extension of food shelf life: (**a**) temperature and humidity sensors in smart packaging; (**b**) applications of smart packaging shelf-life extension.

Extending the shelf life of food products through smart packaging technologies represents a confluence of material science, chemistry, and sensor integration aimed at slowing or halting degradation pathways while maintaining desirable sensory qualities. Building upon the environmental monitoring capacities, these systems employ active and intelligent features to control microenvironmental factors such as oxygen availability, moisture content, ethylene concentration, and temperature stability (Figure 5a,b).

One well-established mechanism for prolonging shelf life is controlling oxidative processes through oxygen scavengers embedded within packaging films or added as sachets. These scavengers, often based on iron powder oxidation or enzymatic reactions, remove residual oxygen from sealed environments, thereby delaying lipid rancidity in high-fat foods and color loss in fresh meats [153,154]. Advanced scavenger formulations incorporate nanomaterials, such as titanium dioxide or zinc oxide particles, that participate in controlled photocatalytic reactions under specific wavelengths, further enhancing the efficiency of oxygen removal. For foods in which partial oxygen is required (e.g., fresh vegetables reliant on aerobic respiration), scavenger capacity is finely tuned to avoid anaerobic conditions that could cause off flavors [114,116]. Controlling moisture is similarly crucial for maintaining the shelf life of a product. Mold growth and altered texture profiles in baked goods can be caused by excessive humidity, whereas extremely low humidity leads to desiccation. Moisture-absorbing inserts or layers within multilayer films help control the relative humidity inside a package. Humectants such as sorbitol and fructose, dispersed within biodegradable polymers, absorb moisture without leaving behind any residues [109,155]. Pairing these absorbers with real-time humidity sensors enables the adaptive release of desiccants only when the levels exceed the threshold values, thereby reducing the use of additives while maintaining optimal textural properties. Ethylene management provides another avenue for shelf-life extension, especially for climacteric fruits whose ripening rate strongly correlates with ethylene concentration [73,86,156]. Active packaging solutions embed ethylene absorbers, such as potassium permanganate supported on inert substrates, within films to retard over-ripening during storage and transport. Intelligent sensors detecting rising ethylene levels can trigger consumer-facing indicators alerting them to peak ripeness; such capability allows retail managers to adjust stock rotation before quality declines irreversibly [29,111]. Plant-derived essential oils (e.g., cinnamon oil rich in cinnamaldehyde) integrated into biodegradable films exhibit targeted inhibition against *Listeria monocytogenes* while slowing lipid oxidation via natural antioxidant action. The multifunctionality achieved by combining antimicrobial emission with oxidative stability reinforcement aligns shelf-life extension goals with consumer preferences for “clean-label” preservative strategies [99,147]. Hybrid designs integrating active preservation agents with intelligent detection modules enhance effectiveness by synchronizing protective actions with environmental monitoring outputs. For instance, CO_2_ emitters used to inhibit bacterial growth in MAP fish filets can be coupled with gas sensors that track headspace concentrations over time; deviations from setpoints may indicate leakage or unexpectedly high respiration rates requiring timely intervention [100]. Similarly, pH-sensitive dyes embedded in films can visually reveal protein breakdown when nitrogenous volatiles increase during seafood storage, alerts that complement microbial inhibition measures already active within the same package. Material selection strongly intersects with the shelf-life capabilities. Chitosan based edible coatings not only act as semi-permeable barriers that moderate gas exchange but also possess intrinsic antimicrobial properties owing to positive charge interactions with microbial cell membranes [98,151,157,158].

Nanocomposite reinforcements using nanoclays or graphene oxide within biopolymer films significantly reduce permeability to O_2_ and H_2_O vapor while enhancing mechanical durability, which is crucial for extended transportation cycles, without compromising barrier integrity. When these functional enhancements occur within compostable substrates, sustainable frameworks gain momentum, meeting environmental targets without sacrificing preservation performance [29,123]. The integration of TTIs serves two purposes for temperature-sensitive products, such as dairy or meat products: validating cold chain adherence and enabling dynamic estimation of the remaining shelf life based on cumulative thermal exposure [96]. The internal microbiological environment of the packaging also benefits from reactive components combining scavengers and inhibitors simultaneously targeting multiple spoilage vectors. Multifunctional nanoparticles, for example, magnesium oxide, provide antifungal effects while regulating moisture, deliver broad-spectrum defense against various degradation routes. Their deployment often reduces dependence on synthetic preservatives applied directly on food surfaces, meeting health-conscious consumer expectations without diminishing storage resilience [3,87,110,119]. Investments in sensor arrays or multifunctional film production need to compensate for gains through reduced spoilage rates across a sufficient volume of turnover before the industry is widely penetrated. Real-world applications highlight how customized solutions surpass generalized shelf-life extension techniques by being accurately matched to specific product requirements. Vacuum-packed sliced ham monitored via ruthenium dye-based O_2_ sensors showed accurate oxygen profiling over time, closely aligning with destructive headspace analysis results, a validation strengthening confidence that intelligent monitoring effectively augments traditional preservation methods [31,41,122]. Similarly, cellulosic packets loaded with silver nanoparticles extended vegetable longevity by inhibiting Aeromonas hydrophile proliferation post-harvest, a pathogen-specific solution that directly targets the produce sector challenge directly. Future research aims at harmonizing regulatory safety thresholds concerning active agents, including migration limits, with performance imperatives ensuring extended shelf life under realistic logistical stresses across diverse geographies [118,147].

### 5.2. Consumer Interaction and Engagement

Consumer interaction and engagement in smart packaging focus on enhancing the user experience through interactive labels, real-time information, and digital tools that promote transparency, trust, and informed decision-making.

**Figure 6:**
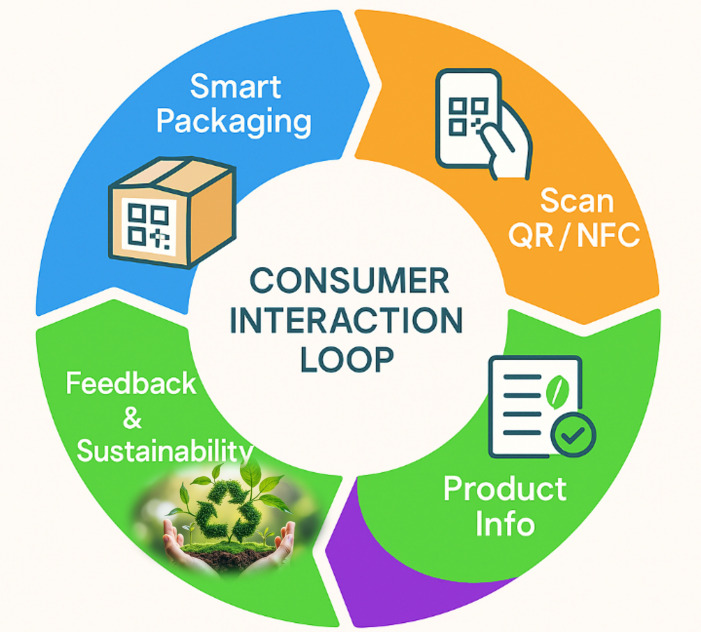
Interactive labels and QR codes.

Interactive labels and QR codes are key tools for increasing smart packaging consumer engagement. They provide quick access to product information, personalized services, and two-way communication between brands and consumers. These digital elements transform static packages into interactive touchpoints, where decisions are guided by real-time, accurate data [159]. In practice, interactive labels range from simple scannable codes to more advanced augmented reality (AR) or near-field communication (NFC) systems (Figure 6).

Among them, QR codes are the most common among them because they are inexpensive, easy to print, and compatible with smartphones. Scanning a QR code gives access to details such as product origin, nutritional data, allergen alerts, and disposal instructions based on local recycling systems.

Sustainability guidance is a major advantage of these labels. QR-linked applications can identify material types at disposal, thereby reducing recycling contamination and improving recovery efficiency. Some systems provide location-based recycling instructions, which help users manage waste correctly despite regional differences [28,100,160,161].

Interactive labels can also enhance food safety. They can connect to cloud-stored freshness indicators or sensor data, which display whether cold chain conditions were upheld. Colorimetric sensors change color in response to temperature fluctuations and relay data via linked QR platforms [36,91,162]. Establishing consumer trust helps prevent the early disposal of food. In addition to quality assurance, interactive packaging can enhance brand engagement.

AR-triggered labels can display recipes, videos, or brand stories. QR scans can offer loyalty points or personalized discounts, transforming simple purchases into continuous interactions. Such tools help brands stand out in competitive markets. From a logistics perspective, QR codes support traceability. Each code serves as a unique identifier linked to blockchain-based records that track distribution and authenticity. Retailers can verify shipments, detect counterfeits, and confirm handling standards without opening packages. However, effective implementation requires attention to accessibility and clarity. Too much promotional content can distract from data on safety or quality. Interfaces must be concise, explaining what users gain from scanning. Visible indicators or printed summaries of sensor data ensure inclusiveness for audiences less familiar with technology.

Ensuring security is a high priority. QR codes can be vulnerable to exploitation if they are altered during manufacturing or if fake versions are created afterwards. Utilizing encrypted or dynamic URLs and secure servers minimizes this risk. NFC tags offer enhanced protection as chip data is more difficult to replicate [163,164]. Strong data integrity safeguards are essential for sensitive products, particularly those with allergen risks, to ensure both consumer safety and protect brand reputation.

Economic considerations also influence design choices. Printing QR codes are low-cost, whereas NFC chips allow richer, automated interactions but are more expensive. Many companies adopt a phased strategy—starting with printed codes for basic engagement and later integrating NFC features for premium lines as ROI becomes clear [165]. Regulations increasingly shape how interactive labels are used. Rules specify minimum font sizes and contrast for scannable reliability, as well as backup printed safety information in case scans fail. Lack of global harmonization forces brands to adapt designs per region, raising costs but ensuring compliance.

Recent research shows the potential of smart integration combining sensors and QR systems. For example, gas sensors that detect spoilage can trigger QR-linked updates that notify retailers or offer discounts before quality declines. Some eco-labels directly link to carbon footprint data or verified offset programs, turning packaging into a channel for environmental action [1,54,166].

Consumer response depends on perceived usefulness. People engage more when scanning offers practical value, such as safety information or recycling help, rather than intrusive marketing. Effective designs balance transparency with optional interactive features. Overall, interactive labels and QR codes bridge the gap between consumers and smart packaging technologies. By directly linking sensor intelligence, traceability data, and sustainability instructions to the user, they combine health protection with environmental responsibility, which are two core goals that shape modern food packaging innovation.

## 6. Impact of Antimicrobial and Antioxidant Nanomaterials on Food Safety and Shelf-Life

In recent years, the development of smart and active food packaging has increasingly relied on nanomaterials not only as structural or barrier enhancers, but also as bioactive components that provide antimicrobial and antioxidant functions. These functionalities directly contribute to reduced microbial spoilage, delayed oxidation, and extended shelf life of food. Beyond classical inorganic nanoparticles such as silver or zinc oxide, emerging quantum materials (e.g., carbon dots) and metal, organic frameworks (MOFs) have attracted attention owing to their tunable structure, large specific surface area, and ability to host or generate bioactive species [130,167].

1. Antibacterial mechanisms and packaging efficacy

Antibacterial nanomaterials typically act through a combination of: (i) disruption of cell membranes, (ii) generation of reactive oxygen species (ROS), (iii) release of toxic ions, and (iv) interaction with intracellular components (proteins, DNA). For instance, silver nanoparticles and ZnO nanoparticles are known to adhere to bacterial cell walls, disturb membrane integrity, and promote leakage of intracellular constituents; in parallel, released metal ions and ROS damage DNA and essential enzymes, ultimately causing cell death [168]. A similar behavior has been demonstrated for carbon dots (CDs), which are a class of carbon-based quantum nanomaterials with sizes typically <10 nm. CDs usually bear abundant surface functional groups (–NH_2_, –OH, –COOH, C=O), enabling both electrostatic interaction with microbial membranes and direct radical scavenging or ROS generation, depending on their chemistry and environment.

Riahi et al. (2025) synthesized carbon dots from red pepper waste and incorporated them into a sodium alginate/gelatin (SA/Gel) matrix to develop bioactive composite films for fruit preservation [130,131]. The authors reported that the red pepper-derived carbon dots (RP-CDs were quasi-spherical (~2.5 nm) and rich in functional groups such as –NH_2_, –OH, C=O, C–O, and C–O–C, which facilitated strong interactions with the biopolymer matrix and, importantly, antibacterial activity. Loading 1–3 wt% RP-CDs into the SA/Gel films not only significantly improved the mechanical strength and moisture resistance but also endowed the films with high bactericidal activity against *Listeria monocytogenes*, reaching 99.9% reduction at 3 wt% CDs [130]. This strong antibacterial effect is attributed to the physical interaction of CDs with bacterial membranes, leading to structural disruption, oxidative stress, and possible interference with cellular components induced by the reactive surface groups of the CDs. The same study demonstrated that the SA/Gel/RP-CD 3% film maintained the freshness of packaged grapes and extended their storage life to 24 days, clearly demonstrating the efficacy of quantum-dot-type nanomaterials in real food packaging scenarios.

Similar results were obtained by other authors. Fu et al. (2022) incorporated green-synthesized CDs into a gelatin/chitosan blend and developed a multifunctional bio-nanocomposite film [169]. The Gel/Chitosan/CDs films exhibited excellent antibacterial activity against spoilage bacteria and were successfully applied as packaging for fish meat, where they reduced quality loss and extended shelf life compared to control films without CDs [169]. These results confirm that carbon-dot-based quantum materials can act as both structural reinforcements and active antibacterial agents in perishable food packaging.

Metal–organic frameworks (MOFs), particularly Zn-based MOFs such as ZIF-8, have emerged as another class of advanced nanomaterials for active packaging. Riahi et al. (2023) engineered high-performance gelatin-based films integrated with Zn-MOFs (ZIF-8) and showed that MOF-loaded films possessed strong antibacterial activity against both Gram-positive (*Staphylococcus aureus*, *L. monocytogenes*) and Gram-negative (*Salmonella enterica*, *Escherichia coli*) bacteria [170]. The authors explained that the porous MOF structure allows for controlled interaction and, potentially, controlled release of Zn^2+^ ions, which can disrupt bacterial membranes, interfere with enzymatic systems, and induce oxidative stress in microbial cells. Applying these Gel/Zn-MOF films were used to package cherry tomatoes, they effectively inhibited microbial growth and retarded texture deterioration over 16 days of storage at 10 °C, demonstrating their practical antimicrobial efficacy in a real food system.

2. Antioxidant mechanisms and oxidative stability

The antioxidant performance of emerging nanomaterials plays a crucial role in enhancing the oxidative stability of active food packaging systems. Lai (2022) reported that polymeric antioxidant films enriched with nanoscale bioactive fillers in lipid-rich food matrices significantly reduced lipid oxidation and delayed rancidity, highlighting the potential of nanostructures as radical scavengers [171]. Carbon-based quantum materials have received increasing attention as multifunctional antioxidant agents. Wu et al. (2025) demonstrated that biodegradable films containing carbon dots derived from agricultural residues exhibited strong reactive oxygen species (ROS) scavenging capacity and provided effective UV shielding, thereby preventing the photo-oxidation of packaged foods [126].

Carrasco et al. (2025) developed poly (vinyl alcohol)–cellulose–MOF composite films with enhanced oxidative stability due to the radical-scavenging behavior of metal–organic frameworks, which helped preserve food quality over extended storage periods [172].

A comprehensive review by Muthu et al. (2025) showed that a wide range of nanomaterials, including metal nanoparticles, nanoclays, nanocellulose and graphene-derived structures are actively being investigated for their antimicrobial, antioxidant and oxygen- or moisture-barrier performance, making them promising candidates for next-generation sustainable food packaging systems [173].

Emerging nanomaterials offer valuable opportunities to delay spoilage and significantly extend the shelf-life of perishable foods.

## 7. New Directions and Innovations for the Development of Sustainable Food Packaging Systems

Advances in artificial intelligence, nanomaterials, and IoT connectivity drive future directions and innovations in smart packaging, paving the way for highly adaptive, sustainable, and intelligent systems that transform how food is monitored, protected, and experienced.

### 7.1. Integration of IoT and AI in Next-Generation Smart Packaging

**Figure 7:**
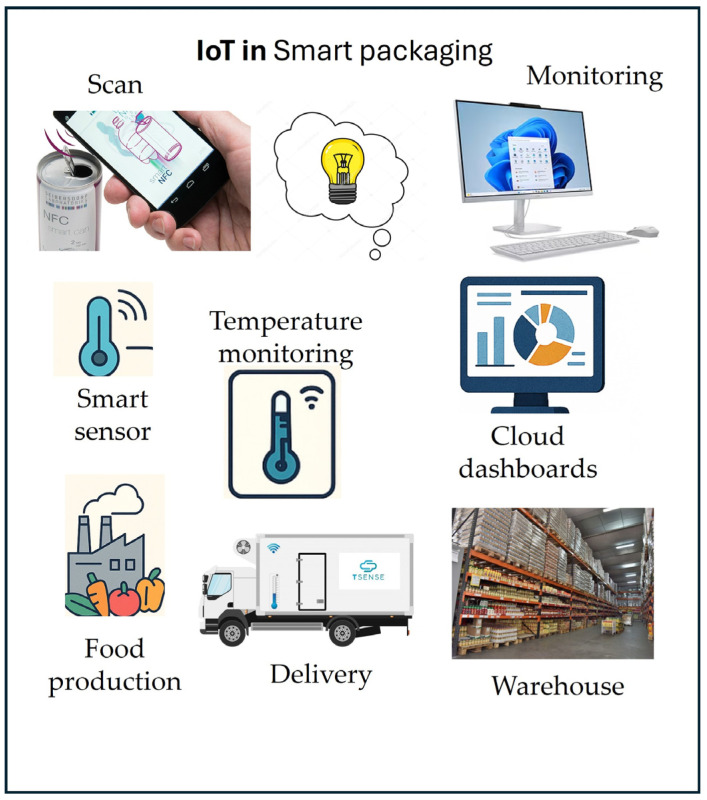
Artificial Intelligence and Internet of Things (IOT) in packaging data analytics.

The integration of Internet of Things (IoT) technologies transforms smart packaging from isolated monitoring units into interconnected components within complex digital ecosystems. Through IoT, each package becomes an active data node capable of communicating real-time information about product condition, transport environment, and origin across the entire food supply chain (Figure 7).

The application of artificial intelligence (AI) to packaging data analytics signifies an advanced point in the development of intelligent food packaging systems. Artificial intelligence transforms heterogeneous data streams into actionable insights by compiling sensor-generated data on temperature, humidity, gas composition, colorimetric indicators and traceability records [165,174].

Embedded sensors within packaging; measuring temperature, humidity, gases, or microbial contamination, continuously transmitting data to cloud-based platforms using RFID, NFC, Bluetooth Low Energy, or cellular networks. These data streams are automatically analyzed by advanced systems that assess product quality, remaining shelf life, and food safety compliance. Some packages employ passive RFID tags, which harvest energy from reader signals, while active tags contain small batteries that enable continuous monitoring, crucial for highly perishable goods such as seafood or dairy products. The transmitted information is secured through encrypted communication channels that comply with international data protection regulations [96,137,175,176].

Bi-directional communication is a major advantage of IoT integration. Packaging sends data and receives operational commands based on network-level risk assessments, such as activating antimicrobial compounds or adjusting scavenger release rates.

Full traceability and authenticity can be achieved by connecting smart packaging to IoT and blockchain-based registries. Each product receives an immutable digital history that records every environmental fluctuation throughout its distribution. Consumers can verify real-time freshness and origin simply by scanning QR or near-field communication (NFC) tags on the package surface [30,41,177].

IoT provides significant operational benefits to supply chain operators. Continuous monitoring enables distributors to reroute shipments showing early signs of deterioration to nearby retail points, thereby reducing waste and financial loss. Meanwhile, AI-powered systems optimize transportation routes, improving efficiency and reducing emissions.

At the consumer level, IoT-connected packages offer complete transparency. Scanning a smart label gives instant access to information on freshness, carbon footprint, and supply chain performance. Some systems even link to loyalty programs, rewarding customers for purchasing well-handled or sustainably sourced goods, thereby strengthening trust and brand engagement [178,179].

However, technical challenges remain. Maintaining stable connectivity in refrigerated or metallic environments, preventing data interception, and managing power efficiency in active devices are key concerns. Edge computing can reduce bandwidth demand and latency by processing data locally before cloud transmission. Global standardization of communication protocols is also essential to ensure seamless interoperability among producers, distributors, customs, and retailers [180,181].

IoT integration promotes sustainability from an environmental standpoint by reducing premature food disposal. Instead of relying on static “best-before” dates, real-time freshness data allow accurate consumption window definition, reducing carbon emissions and minimizing avoidable waste. Recent developments in biodegradable electronics, such as compostable conductive films, further align IoT packaging innovations with eco-regulatory goals [145,182].

Embedding IoT within smart packaging transforms it into an active participant in the digitalized food ecosystem. These packages intelligently monitor, communicate, and respond to environmental changes, ensuring safety, transparency, and sustainability throughout the supply chain. Thus, each package becomes both a guardian of food integrity and a node of collective intelligence, contributing to a more efficient, transparent, and responsible global food system.

### 7.2. Advanced Strategies for Sustainable Food Packaging Development

The transition to sustainable food packaging is a critical priority driven by escalating concerns over plastic pollution and the circular economy principles. Recent literature highlights a paradigm shift toward bio-based materials, smart packaging technologies, and integrated circular economy design strategies.

Future research should concentrate on further improving sensor accuracy, enhancing the biodegradability of packaging materials, and increasing cost-effectiveness to support large-scale, global implementation [145,174,183,184].

**Figure 8:**
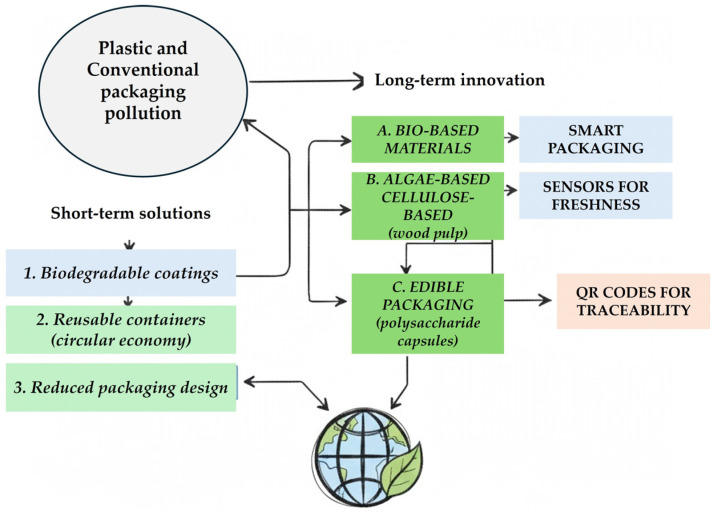
New directions for sustainable food packaging.

New directions in smart packaging must prioritize improving the accuracy and long-term stability of biosensors and intelligent indicators to enable more reliable real-time food monitoring (Figure 8).

Advances in nanotechnology and AI-based predictive models are expected to further refine food safety evaluation and quality prediction. Moreover, developing cost-efficient and scalable biodegradable packaging concepts is essential to ensure economic feasibility without compromising functional performance. Furthermore, large-scale pilot implementations must demonstrate commercial viability under real supply-chain conditions.

Finally, gaining insights into consumer perception and strengthening public awareness through targeted educational strategies will be critical for the broader societal acceptance of smart packaging technologies.

## 8. Conclusions

Smart packaging marks a major transformation in food preservation, safety, and sustainability, achieved by merging advanced materials, sensor technologies, and digital connectivity. These systems actively monitor and respond to environmental changes, extending shelf life, reducing waste, and strengthening consumer trust. The shift from passive to intelligent packaging aligns functionality with environmental responsibility, using biodegradable materials and nanotechnology to balance performance and sustainability. Integrated temperature, humidity, and gases sensors enable continuous monitoring, whereas interactive indicators and data carriers improve traceability and safety across supply chains.

Consumer engagement grows through interactive labels and transparent product information, although success depends on clear communication, trust, and privacy protection. Economic and regional factors influence adoption, whereas life-cycle assessments emphasize the need for recyclable and compostable designs.

The remaining challenges include sensor stability, system calibration, and compliance with evolving legal and safety standards. Progress in AI and IoT will further enhance predictive quality control, logistics optimization, and autonomous packaging responses. Ultimately, smart packaging emerges as a cornerstone of sustainable food systems, combining innovation with responsibility.

The significance of this study lies in its comprehensive examination of the latest advances and persistent challenges in smart packaging technologies for the food industry, offering critical insights into how emerging materials, sensor integration, and data-driven analytics can collectively enhance food safety, extend shelf life, and support sustainable supply chain management in alignment with global food security and environmental objectives.

## Figures and Tables

**Table 1:** Smart packaging applications in food industry.

(a). Meat and Meat Products		
Application Focus	Reported and Quantified Outcome	References
Absorbent pads with lemongrass essential oil integration	Enhanced inhibition zones against *S. aureus* and *E. coli* compared to control samples	[49,50,51]
Blockchain -based supply chain records linked with intelligent labels	Improved transparency and consumer trust through farm-to-retail traceability data access	[52,53]
Natural antimicrobial active films	Met consumer preference for additive-free preservation while aligning with sustainability targets	[53]
Absorbent pad + lemongrass essential oil under MAP conditions	1–1.5 log CFU/g reduction viable count over 12 days of chilled storage compared to control packs; maintained acceptable color scores	[49,54]
Silver-zeolite LDPE film wrap for fresh beef cuts	Inhibited mesophilic bacteria growth by 2–3 log CFU/g over 14 days at 4 °C while sustaining lipid oxidation below threshold PVs	[55,56]
PHMB-loaded nanofiber mats placed inside overwrap trays for pork slices	Reduced aerobic plate count growth rate constant k by ≈35% relative to controls; no degradation of drip loss performance parameters	[57]
Volatile amine-sensitive freshness indicator label integrated into high- $O_{2}$ MAP beef steaks	Provided visual alert (>90% correct positive signals) prior to reaching the sensory rejection thresholds determined by trained panelists	[58,59]
RFID-linked gas-sensing MAP containers for export lamb shipments (>20 days transit)	Continuous $O_{2}$/ $CO_{2}$ logging identified micro-leakage events allowing corrective action before sensory deterioration; reduced shipment rejections by ≈15% year-on-year	[60]
(b). Milk and dairy products		
Application focus	Reported and quantified outcome	References
Embedded biosensors detecting lactic acid accumulation	Early warning system for microbial growth enabling timely removal from distribution channels	[58,61]
Intelligent indicators monitoring temperature fluctuations during transport/storage	Provided verifiable cold-chain compliance data to retailers and consumers	[55,62]
Bio-nanocomposite films improving barrier properties against oxygen ingress	Reduced oxidation-related flavor deterioration over longer storage periods	[58]
Chitosan –PLA composite film wrap for soft cheese	Suppressed surface bacterial counts by >2 log CFU/cm^2^ over 10-day storage at 4 °C; no effect on pH or ripening profile	[63]
O _2_ scavenging bottle closures for UHT milk	Reduced headspace oxygen from 1.8% to <0.05% within three days; prevented oxidative off-flavors up to end-of-shelf-life testing point at 120 days ambient storage	[64]
Lactic acid biosensor integrated into biodegradable multilayer carton liners	Provided early spoilage alert ≈48 h before sensory detection threshold; maintained full compostability under industrial conditions post-use	[58,65]
TTI label on HDPE fresh milk bottles in urban chilled distribution trials	Correctly identified 95% of cold-chain breaks exceeding 2 h at >8 °C during monitored delivery routes over two months study period	[66]
RFID-linked TTI arrays in export butter shipments (>30 days) logging continuous temperature profiles accessible via blockchain nodes at import inspection points	Enabled rejection avoidance by re-validating compliance in cases where container sensor logs contested portside infractions claims; reduced unjustified discard incidents by ≈12% year-on-year	[67]
(c). Bakery products		
Application focus	Reported and quantified outcome	References
PLA –protein composite film with clove oil for sliced bread packaging	Reduced visible mold incidence by 80% over 10-day ambient storage; no adverse sensory defect noted	[68]
Starch-based laminate incorporating anhydrous calcium chloride layer for cookies transport in humid climates	Maintained $a_{w}$ below 0.50 during 30-day test cycle; prevented loss of crispness relative to unprotected controls	[67,69]
Rosemary-extract infused LDPE pouches for butter-rich biscuits	Delayed peroxide value (PV) increase beyond sensory detection threshold by ≈40% over 60 days storage at 25 °C	[55,70]
Humidity-sensitive printed ink patch inside bread bag headspace area indicating unsafe high-moisture condition via irreversible color change	Correctly forecast mold outbreak risk 24–48 h before visual mycelium appearance on loaf crusts under simulated distribution conditions	[58,71]
Compostable cellulose-acetate cupcake liners impregnated with cinnamon oil vapors release system	Extended microbial-free shelf life by ≈3 days under bakery store display settings while enabling post-use biodegradation within 90 days under industrial composting conditions	[69]
(d) Vegetables and Fruits		
Application focus	Reported and quantified outcome	References
Activated carbon -clay composite film for bananas during shipping	Extended green stage by ≈9 days relative to plain PE wrap; maintained firmness within quality threshold for retail display acceptance rates above 85% after arrival	[72]
Cellulose fiber-based humidity absorber pads for packaged lettuce leaves	Reduced mass loss from turgor decline by 22% over 7-day storage; microbial counts remained below spoilage limit throughout trial period	[53,69]
Graphene oxide VOC sensor arrays integrated into strawberry export clamshells	Achieved >90% predictive accuracy for mold development within 48 h of first detectable colony growth under partial refrigeration transport regimes	[55,73]
PLA –chitosan biodegradable films with essential oil microcapsules for cherry tomatoes	Suppressed visible spoilage incidence by ≈40% after 14 days ambient storage compared with non-active PLA controls; maintained average lycopene content retention above 95% baseline level measured at harvest day	[74]
Multi-layer sachet system combining potassium permanganate granules and silica-gel desiccant for mixed-fruit cartons during intercontinental sea freight (>21 days)	Reduced ethylene concentration inside carton headspace from initial 0.45 ppm to below 0.10 ppm throughout transit; prevented cross-ripening chain reaction among mixed climacteric loads leading to rejection rate drop from 12% to under 5% year-on-year shipment statistics	[55,75]

**Table 2:** Classification of smart packaging types applied to food industry.

(a). Meat and Meat Products		
Type	Description	References
Passive	High-barrier multilayer films prevent oxygen/moisture ingress; vacuum-sealed PE or PP formats for chilled transport	[76]
Active	Antimicrobial films with essential oils or silver-zeolites; oxygen scavengers integrated into modified atmosphere packaging (MAP) units	[55,77]
Intelligent	Volatile amine detectors: pH-sensitive indicators signaling microbial activity before sensory spoilage markers arise	[58,78]
Hybrid Smart Systems	MAP with embedded gas sensors transmitting data via RFID for continuous cold chain surveillance	[69]
(b). Milk and dairy products		
Type	Description	References
Passive	High-barrier cartons or multilayer bottles minimizing light and oxygen ingress; PET with UV-blocking additives for milk	[79,80]
Active	Antimicrobial films/coatings (e.g., chitosan-based) inhibiting psychrotrophs; oxygen scavenging closures for cheese ripening control	[55,81]
Intelligent	TTIs signaling cumulative chill-chain breaks; biosensors detecting pH or lactic acid change indicating microbial growth	[66]
Hybrid Smart Systems	RFID-enabled TTIs logging temperature history linked with blockchain for cold chain audits in export milk consignments	[69,82]
(c). Bakery products		
Type	Description	References
Passive	High-barrier laminates preventing moisture ingress; metallized films blocking light-induced oxidation in chocolate coatings	[69]
Active	Moisture absorbers embedded in bread bags; antifungal essential oil-infused biopolymer film wraps	[55,83]
Intelligent	Humidity-sensitive inks alerting excessive water vapor for mold-risk scoring; integrity seals showing tamper detection	[58,84]
Hybrid Smart Systems	Bio-based films combining humidity absorption with visual indicator windows color-coded for safe/unsafe conditions	[79,85]
(d). Vegetables and Fruits		
Type	Description	References
Passive	Gas-permeable films allowing controlled respiration while limiting water vapor transmission; UV-blocking wraps to prevent photodegradation of pigments	[69]
Active	Ethylene scavenger sachets; humidity absorber pads preventing fungal proliferation in high-moisture items	[55,86]
Intelligent	VOC-sensitive nanosensors for senescence detection; TTI labels recording temperature deviations during long-haul shipping	[58,87]
Hybrid Smart Systems	Biodegradable films incorporating both ethylene absorption agents and real-time freshness indicator windows visible to handlers/consumers	[87]

**Table 3:** Benchmark packaging systems: comparative characteristics, functional performance, and indicative cost considerations.

Packaging System Type	Key Characteristics/Typical Performance	Relevance as a Benchmark for Smart Packaging	Indicative Cost and Scalability Considerations	References
Conventional passive packaging (plastics, paper, glass, metal)	Provides basic barriers and protection; no active or sensing functions	Serves as the traditional baseline for evaluating the added value of active/smart systems	Generally low cost due to mass production and established industrial supply chains; highly scalable	[7,10,17,152]
Active packaging systems	Includes antimicrobial/antioxidant agents; oxygen/moisture scavengers	Benchmark for functional improvement over passive packaging	Moderate material and incorporation cost depending on active agents; scalable but sometimes limited by regulatory approval of active substances	[10,16,17,150]
Intelligent monitoring packaging (indicators, sensors, RFID, TTIs)	Provides real-time information on product quality or temperature history	Benchmark for evaluating intelligence and monitoring capability	Higher cost due to sensing elements, electronics or smart indicators; scalability depends on target food category and cost–benefit ratio	[17,149,150]
Nano-enabled active and intelligent packaging (nanocomposites, nano-coatings, nano-sensors)	Enhanced barrier, antimicrobial/antioxidant functionality, possible sensing capabilities; often multifunctional	Represents an advanced benchmark for high-performance smart packaging	Costs depend on nanomaterial type, synthesis method, loading level, and regulatory requirements; industrial scale-up feasible but still under development for certain nano-platforms	[10,17,149,152]

## Data Availability

No new data were created or analyzed in this study. Date sharing is not applicable.

## References

[1] Peng W., Peng H. (2024). Green food packaging industry to explore and analyze. Int. J. Nat. Resour. Environ. Stud..

[2] Hassoun A. (2025). Food sustainability 4.0: Harnessing fourth industrial revolution technologies for sustainable food systems. Discov. Food.

[3] Zubair M., Rauf Z., Fatima S., Ullah A. (2024). Lignin-derived bionanocomposites as functional food packaging materials. Sustain. Food Technol..

[4] Waldhans C., Albrecht A., Ibald R., Wollenweber D., Sy S.J., Kreyenschmidt J. (2024). Temperature control and data exchange in food supply chains: Applicability of digitalized time–temperature indicators for cold chain optimization. J. Packag. Technol. Res..

[5] Young E., Mirosa M., Bremer P. (2020). A systematic review of consumer perceptions of smart packaging technologies for food. Front. Sustain. Food Syst..

[6] Clark N., Trimingham R., Storer I. (2019). Understanding the views of the UK food packaging supply chain to support a move to circular economy systems. Packag. Technol. Sci..

[7] González-López M.E., Calva-Estrada S.J., Gradilla-Hernández M.S., Barajas-Álvarez P. (2023). Current trends in biopolymers for food packaging: A review. Front. Sustain. Food Syst..

[8] Petrenko L., Puzko S., Lavrenenko V., Gernego I. (2024). Fostering sustainable packaging industry: Global trends and challenges. Eur. J. Sustain. Dev..

[9] Mazumder S., Chanda S., Bhaumik A. (2022). Active and intelligent packaging technologies: An aspect of food safety management. Int. J. Curr. Res. Rev..

[10] Balasubramanian M., Chellasudheer M., Manojkumar V., Arasu J., Mathesh T.A. (2023). New advances in smart packaging production methods. Int. J. Trendy Res. Eng. Technol..

[11] Huang X. (2023). Blockchain-enabled smart packaging: Enhancing food traceability and consumer confidence in the Chinese food industry. Front. Soc. Sci. Technol..

[12] Mousavi M.M., Mahmoudpour M. (2024). Function and application of some active and antimicrobial packaging in the food industry: A review. J. Microbiota.

[13] Lamri M., Bhattacharya T., Boukid F., Chentir I., Dib A.L., Das D., Djenane D., Gagaoua M. (2021). Nanotechnology as a processing and packaging tool to improve meat quality and safety. Foods.

[14] Albrecht A., Ibald R., Raab V., Reichstein W., Haarer D., Kreyenschmidt J. (2020). Implementation of time–temperature indicators to improve temperature monitoring and support dynamic shelf life in meat supply chains. J. Packag. Technol. Res..

[15] Ceballos R.L., Ochoa-Yepes O., Goyanes S., Bernal C., Famá L. (2020). Effect of yerba mate extract on the performance of starch films obtained by extrusion and compression molding as active and smart packaging. Carbohydr. Polym..

[16] Yüceer M., Caner C. (2023). Intelligent packaging and applications in the food industry. J. Food Feed Sci. Technol..

[17] Salgado P.R., Di Giorgio L., Musso Y.S., Mauri A.N. (2021). Recent developments in smart food packaging focused on biobased and biodegradable polymers. Front. Sustain. Food Syst..

[18] Qazanfarzadeh Z., Masek A., Chakraborty S., Kumaravel V. (2024). Development of brewer’s spent grain-derived bionanocomposites through a multiproduct biorefinery approach for food packaging. Ind. Crops Prod..

[19] Yang Z., Zhai X., Zou X., Shi J., Huang X., Li Z., Gong Y., Holmes M., Povey M., Xiao J. (2021). Bilayer pH-sensitive colorimetric films with light-blocking ability and electrochemical writing property: Application in monitoring crucian spoilage in smart packaging. Food Chem..

[20] Chen S., Brahma S., Mackay J., Cao C., Aliakbarian B. (2020). The role of smart packaging systems in the food supply chain. J. Food Sci..

[21] D’Almeida A.P., de Albuquerque T.L. (2024). Innovations in food packaging: From bio-based materials to smart packaging systems. Processes.

[22] Runtuk J.K., Maukar A.L. (2019). Analysis and framework for agricultural supply chain improvement: A case study of California papaya in Cikarang. J. Sist. Manaj. Ind..

[23] Kim E. (2019). Development of hybrid food products safety control technology and green supply chain management (GSCM): Theory and design. Alanya Acad. Rev..

[24] Alamri M.S., Qasem A.A., Mohamed A.A., Hussain S., Ibraheem M.A., Shamlan G., Hesham A.A., Qasha A.S. (2021). Food packaging’s materials: A food safety perspective. Saudi J. Biol. Sci..

[25] Tripathi G., Ahad M.A., Casalino G. (2023). A comprehensive review of blockchain technology: Underlying principles and historical background with future challenges. Decis. Anal. J..

[26] Stanciu A.C. (2024). Smart packaging main trends. Ovidius Univ. Ann. Econ. Sci. Ser..

[27] Poli M., Malagas K., Nomikos S., Papapostolou A., Vlassas G. (2023). An overview of the impact of the food sector “intelligent packaging” and “smart packaging”. Eur. J. Interdiscip. Stud..

[28] Müller P., Schmid M. (2019). Intelligent packaging in the food sector: A brief overview. Foods.

[29] Tas C.E., Hendessi S., Baysal M., Unal S., Cebeci F.C., Menceloglu Y.Z., Unal H. (2017). Halloysite nanotubes/polyethylene nanocomposites for active food packaging materials with ethylene scavenging and gas barrier properties. Food Bioprocess Technol..

[30] Ma Y., Yang W., Xia Y., Xue W., Wu H., Li Z., Zhang F., Qiu B., Fu C. (2022). Properties and applications of intelligent packaging indicators for food spoilage. Membranes.

[31] Zhai X., Li Z., Shi J., Huang X., Sun Z., Zhang D.I., Zou X., Sun Y., Zhang J., Holmes M. (2019). A colorimetric hydrogen sulfide sensor based on gellan gum–silver nanoparticles bionanocomposite for monitoring meat spoilage in intelligent packaging. Food Chem..

[32] Realini C.E., Marcos B. (2014). Active and intelligent packaging systems for a modern society. Meat Sci..

[33] Schaefer D., Cheung W.M. (2018). Smart packaging: Opportunities and challenges. Procedia CIRP.

[34] Chia M.R., Ahmad I., Phang S.W. (2022). Starch/polyaniline biopolymer film as potential intelligent food packaging with colourimetric ammonia sensor. Polymers.

[35] Karmaus A.L., Osborn R., Krishan M. (2018). Scientific advances and challenges in safety evaluation of food packaging materials: Workshop proceedings. Regul. Toxicol. Pharmacol..

[36] Dirpan A., Djalal M., Ainani A.F. (2022). A simple combination of active and intelligent packaging based on garlic extract and indicator solution in extending and monitoring meat quality stored at cold temperature. Foods.

[37] Gigauri I., Palazzo M. (2023). Intelligent packaging as a marketing tool: Are digital technologies reshaping packaging?. Agora Int. J. Econ. Sci..

[38] Yele S., Litoriya R. (2024). Blockchain-based secure dining: Enhancing safety, transparency, and traceability in food consumption environments. Blockchain Res. Appl..

[39] Singh A., Gutub A., Nayyar A., Khan M.K. (2023). Redefining food safety traceability system through blockchain: Findings, challenges, and open issues. Multimed. Tools Appl..

[40] Abedi-Firoozjah R., Yousefi S., Heydari M., Seyedfatehi F., Jafarzadeh S., Mohammadi R., Rouhi M., Garavand F. (2022). Application of red cabbage anthocyanins as pH-sensitive pigments in smart food packaging and sensors. Polymers.

[41] Azeredo H.M., Correa D.S. (2021). Smart choices: Mechanisms of intelligent food packaging. Curr. Res. Food Sci..

[42] Won S., Won K. (2021). Self-powered flexible oxygen sensors for intelligent food packaging. Food Packag. Shelf Life.

[43] Kumari S., Debbarma R., Nasrin N., Khan T., Taj S., Bhuyan T. (2024). Recent advances in packaging materials for food products. Food Bioeng..

[44] Fadiji T., Pathare P.B. (2023). Technological advancements in food processing and packaging. Processes.

[45] Flores Y., Pelegrín C.J., Ramos M., Jiménez A., Garrigós M.C. (2021). Use of herbs and their bioactive compounds in active food packaging. Aromatic Herbs in Food.

[46] Calabretta M.M., Gregucci D., Desiderio R., Michelini E. (2023). Colorimetric paper sensor for food spoilage based on biogenic amine monitoring. Biosensors.

[47] Kitz R., Walker T., Charlebois S., Music J. (2022). Food packaging during the COVID-19 pandemic: Consumer perceptions. Int. J. Consum. Stud..

[48] Zafar M.Z., Shi X., Yang H., Abbas J., Chen J. (2022). The impact of interpretive packaged food labels on consumer purchase intention. Int. J. Environ. Res. Public Health.

[49] Dirpan A., Iqbal M., Yumeina D., Prahesti K.I., Djalal M., Syarifuddin A., Bahmid N.A., Bastian F., Salengke S., Suryono S. (2023). Smart packaging for chicken meat quality: Absorbent food pad and Whatman paper applications. Indones. Food Sci. Technol. J..

[50] Lee H., Lee S. (2024). Development of essential oil-containing antimicrobial and deodorizing nanofibrous membranes for sanitary napkin applications. J. Ind. Text..

[51] Drago E., Campardelli R., Pettinato M., Perego P. (2020). Innovations in smart packaging concepts for food: An extensive review. Foods.

[52] Gazzola P., Pavione E., Barge A., Fassio F. (2023). Using the transparency of supply chain powered by blockchain to improve sustainability relationships with stakeholders in the food sector: The case study of Lavazza. Sustainability.

[53] Cordeiro M., Ferreira J.C. (2025). Beyond traceability: Decentralised identity and digital twins for verifiable product identity in agri-food supply chains. Appl. Sci..

[54] Kontominas M.G., Badeka A.V., Kosma I.S., Nathanailides C.I. (2021). Recent developments in seafood packaging technologies. Foods.

[55] Kaushani K.G., Rathnasinghe N.L., Katuwawila N., Jayasinghe R.A., Nilmini A.H.L.R., Priyadarshana G. (2022). Trends in smart packaging technologies for sustainable monitoring of food quality and safety. Int. J. Res. Innov. Appl. Sci..

[56] Shankar S., Bang Y.J., Rhim J.W. (2019). Antibacterial LDPE/GSE/Mel/ZnONP composite film-coated wrapping paper for convenience food packaging application. Food Packag. Shelf Life.

[57] Karanth S., Feng S., Patra D., Pradhan A.K. (2023). Linking microbial contamination to food spoilage and food waste: The role of smart packaging, spoilage risk assessments, and date labeling. Front. Microbiol..

[58] Bhatlawande A.R., Ghatge P.U., Shinde G.U., Anushree R.K., Patil S.D. (2024). Unlocking the future of smart food packaging: Biosensors, IoT, and nanomaterials. Food Sci. Biotechnol..

[59] Sani A.M., Zhang W., Abedini A., Khezerlou A., Shariatifar N., Assadpour E., Zhang F., Jafari S.M. (2024). Intelligent packaging systems for the quality and safety monitoring of meat products: From lab scale to industrialization. Food Control.

[60] Heo W., Lim S. (2024). A review on gas indicators and sensors for smart food packaging. Foods.

[61] Palanisamy Y., Kadirvel V., Ganesan N.D. (2024). Recent technological advances in food packaging: Sensors, automation, and application. Sustain. Food Technol..

[62] Yar M.S., Ibeogu I.H., Regmi A., Zhang N., Li C. (2025). Advances in intelligent time-temperature indicators for cold chain monitoring: Mechanisms, challenges, and applications. Trends Food Sci. Technol..

[63] Barik M., BhagyaRaj G.V.S., Dash K.K., Shams R. (2024). A thorough evaluation of chitosan-based packaging film and coating for food product shelf-life extension. J. Agric. Food Res..

[64] Panzaru C., Radu-Rusu R.M., Dolis M.G., Nistor M., Maciuc V., Davidescu M.A. (2024). Contributions to the study of milk quality from various cattle breeds. SCSCC6.

[65] Sobhan A., Hossain A., Wei L., Muthukumarappan K., Ahmed M. (2025). IoT-enabled biosensors in food packaging: A breakthrough in food safety for monitoring risks in real time. Foods.

[66] Abekoon T., Buthpitiya B.L.S.K., Sajindra H., Samarakoon E.R.J., Jayakody J.A.D.C.A., Kantamaneni K., Rathnayake U. (2024). A comprehensive review to evaluate the synergy of intelligent food packaging with modern food technology and artificial intelligence field. Discov. Sustain..

[67] Karnwal A., Rauf A., Jassim A.Y., Selvaraj M., Al-Tawaha A.R.M.S., Kashyap P., Kumar D., Malik T. (2025). Advanced starch-based films for food packaging: Innovations in sustainability and functional properties. Food Chem. X.

[68] Cooper K.A., Quested T.E., Lanctuit H., Zimmermann D., Espinoza-Orias N., Roulin A. (2018). Nutrition in the bin: A nutritional and environmental assessment of food wasted in the UK. Front. Nutr..

[69] Oboturova N., Povetkin S., Nikulnikova N., Lazareva N., Klopova A., Lyubchanskiy N., Sukhanova E., Lebedeva N. (2024). Upcycling agricultural byproducts into eco-friendly food packaging. Potravinar. Slovak J. Food Sci..

[70] Halim-Lim S.A., Baharuddin A.A., Cherrafi A., Ilham Z., Jamaludin A.A., David W., Sodhi H.S. (2023). Digital innovations in the post-pandemic era towards safer and sustainable food operations: A mini-review. Front. Food Sci. Technol..

[71] Bilgin M., Backhaus J. (2020). Development of an irreversible hydrochromic ink for smart packaging. J. Print Media Technol. Res..

[72] Xie C., Wang F., He Z., Tang H., Li H., Hou J., Liu Y., Jiang L. (2023). Development and characterization of active packaging based on chitosan/chitin nanofibers incorporated with scallion flower extract for fresh-cut bananas. Int. J. Biol. Macromol..

[73] Alam A.U., Rathi P., Beshai H., Sarabha G.K., Deen M.J. (2021). Fruit quality monitoring with smart packaging. Sensors.

[74] Zehra A., Wani S.M., Bhat T.A., Jan N., Hussain S.Z., Naik H.R. (2022). Preparation of biodegradable chitosan packaging film based on ZnO, CaCl_2_, nanoclay and PEG with thyme oil. Int. J. Biol. Macromol..

[75] Wyrwa J., Barska A. (2017). Innovations in the food packaging market: Active packaging. Eur. Food Res. Technol..

[76] Hossain A. (2023). Do date codes cause food waste? Smart packaging might tackle the problem. J. Food Bioact..

[77] Kusuma H.S., Yugiani P., Himana A.I., Aziz A. (2024). Reflections on food security and smart packaging. Polym. Bull..

[78] Kühn D., Profeta A., Krikser T., Heinz V. (2023). Adaption of the meat attachment scale (MEAS) to Germany: Interplay with food neophobia, preference for organic foods, social trust and trust in food technology innovations. Agric. Food Econ..

[79] Pânzaru C., Doliș M.G., Radu-Rusu R.-M., Pascal C., Maciuc V., Davidescu M.-A. (2024). Equine Milk and Meat: Nutritious and Sustainable Alternatives for Global Food Security and Environmental Sustainability—A Review. Agriculture.

[80] Achmadi E.R. (2023). Strategies managing smart packaging for food application. J. Food Agric. Prod..

[81] Kumar S., Mukherjee A., Dutta J. (2020). Chitosan-based nanocomposite films and coatings: Emerging antimicrobial food packaging alternatives. Trends Food Sci. Technol..

[82] Ruippo L., Koivula H., Korhonen J., Toppinen A., Kylkilahti E. (2023). Innovating for sustainability: Attributes, motivations, and responsibilities in the Finnish food packaging ecosystem. Circ. Econ. Sustain..

[83] Safakas K., Lainioti G.C., Tsiamis G., Stathopoulou P., Ladavos A. (2025). Utilizing essential oil components as natural antifungal preservatives in active bread packaging. Polymers.

[84] Mulloni V., Marchi G., Gaiardo A., Valt M., Donelli M., Lorenzelli L. (2024). Applications of Chipless RFID Humidity Sensors to Smart Packaging Solutions. Sensors.

[85] Sun Q., Yuan Y., Xu B., Gao S., Zhai X., Xu F., Shi J. (2025). Innovative technologies reshaping meat industrialization: Challenges and opportunities in the intelligent era. Foods.

[86] Mariah M.A.A., Vonnie J.M., Erna K.H., Nur’Aqilah N.M., Huda N., Wahab R.A., Rovina K. (2022). The Emergence and Impact of Ethylene Scavengers Techniques in Delaying the Ripening of Fruits and Vegetables. Membranes.

[87] Ramezani G., Assadpour E., Zhang W., Jafari S.M. (2025). Carbon nanomaterial-based sensors for smart food packaging. Ind. Crops Prod..

[88] Ghaani M., Cozzolino C.A., Castelli G., Farris S. (2016). An overview of the intelligent packaging technologies in the food sector. Trends Food Sci. Technol..

[89] Dirpan A., Hidayat S.H., Djalal M., Ainani A.F., Yolanda D.S., Khosuma M., Solon G.T., Ismayanti N. (2023). Trends over the last 25 years and future research into smart packaging for food: A review. Future Foods.

[90] Palmieri F., Tagoe J.N.A., Di Maio L. (2024). Development of PBS/nanocomposite PHB-based multilayer blown films with enhanced properties for food packaging applications. Materials.

[91] Said N.S., Lee W.Y. (2025). Pectin-Based Active and Smart Film Packaging: A Comprehensive Review of Recent Advancements in Antimicrobial, Antioxidant, and Smart Colorimetric Systems for Enhanced Food Preservation. Molecules.

[92] Pascall M.A., DeAngelo K., Richards J., Arensberg M.B. (2022). Role and importance of functional food packaging in specialized products for vulnerable populations: Implications for innovation and policy development for sustainability. Foods.

[93] Priyadarshi R., Ezati P., Rhim J.W. (2021). Recent advances in intelligent food packaging applications using natural food colorants. ACS Food Sci. Technol..

[94] Gupta D., Lall A., Kumar S., Patil T.D., Gaikwad K.K. (2024). Plant-based edible films and coatings for food-packaging applications: Recent advances, applications, and trends. Sustain. Food Technol..

[95] Morris M.A., Padmanabhan S.C., Cruz-Romero M.C., Cummins E., Kerry J.P. (2017). Development of active, nanoparticle, antimicrobial technologies for muscle-based packaging applications. Meat Sci..

[96] Li T., Lloyd K., Birch J., Wu X., Mirosa M., Liao X. (2020). A quantitative survey of consumer perceptions of smart food packaging in China. Food Sci. Nutr..

[97] Janssen M., Chang B.P., Hristov H., Pravst I., Profeta A., Millard J. (2021). Changes in food consumption during the COVID-19 pandemic: Analysis of consumer survey data from the first lockdown period in Denmark, Germany, and Slovenia. Front. Nutr..

[98] Ton-That P., Dinh T.A., Gia-Thien H.T., Van Minh N., Nguyen T., Huynh K.P.H. (2025). Novel packaging chitosan film decorated with green-synthesized nanosilver derived from dragon fruit stem. Food Hydrocoll..

[99] Chawla R., Sivakumar S., Kaur H. (2021). Antimicrobial edible films in food packaging: Current scenario and recent nanotechnological advancements. Carbohydr. Polym. Technol. Appl..

[100] Wu D., Zhang M., Chen H., Bhandari B. (2021). Freshness monitoring technology of fish products in intelligent packaging. Crit. Rev. Food Sci. Nutr..

[101] Zhang J., Zou X., Zhai X., Huang X., Jiang C., Holmes M. (2019). Preparation of an intelligent pH film based on biodegradable polymers and roselle anthocyanins for monitoring pork freshness. Food Chem..

[102] Lyn F.H., Ismail-Fitry M.R., Noranizan M.A., Tan T.B., Hanani Z.N. (2024). Recent advances in extruded polylactic acid-based composites for food packaging: A review. Int. J. Biol. Macromol..

[103] Kamboj A., Kaur G., Jain H., Singh L. (2025). The role of smart packaging and consumer psychology in reducing food waste: A review. Food Humanit..

[104] Dodero A., Escher A., Bertucci S., Castellano M., Lova P. (2021). Intelligent packaging for real-time monitoring of food quality: Current and future developments. Appl. Sci..

[105] Cheng M., Yan X., Cui Y., Han M., Wang X., Wang J., Zhang R. (2022). An eco-friendly film of pH-responsive indicators for smart packaging. J. Food Eng..

[106] Lindh A., Wijayarathna E.K.B., Ciftci G.C., Syed S., Bashir T., Kadi N., Zamani A. (2024). Dry gel spinning of fungal hydrogels for the development of renewable yarns from food waste. Fungal Biol. Biotechnol..

[107] Arunan I., Crawford R.H. (2021). Greenhouse gas emissions associated with food packaging for online food delivery services in Australia. Resour. Conserv. Recycl..

[108] Das P.P., Prathapan R., Ng K.W. (2024). Advances in biomaterials-based food packaging systems: Current status and the way forward. Biomater. Adv..

[109] Mustafa F., Andreescu S. (2020). Nanotechnology-based approaches for food sensing and packaging applications. RSC Adv..

[110] Sun X., Li Q., Wu H., Zhou Z., Feng S., Deng P., Zou H., Tian D., Lu C. (2023). Sustainable starch/lignin nanoparticle composite biofilms for food packaging applications. Polymers.

[111] Bhargava N., Sharanagat V.S., Mor R.S., Kumar K. (2020). Active and intelligent ethyladable packaging films using food and food waste-derived bioactive compounds: A review. Trends Food Sci. Technol..

[112] Koreshkov M., Takatsuna Y., Bismarck A., Fritz I., Reimhult E., Zirbs R. (2024). Sustainable food packaging using modified kombucha-derived bacterial cellulose nanofillers in biodegradable polymers. RSC Sustain..

[113] Gao X., Cao L., Wang L., Liu S., Zhang M., Li C., Waterhouse G.I.N., Fan H., Xu J. (2024). Z-scheme heterojunction g-C3N4–TiO2 reinforced chitosan/poly(vinyl alcohol) film: Efficient and recyclable for fruit packaging. Int. J. Biol. Macromol..

[114] Lawal U., Kumar N., Samyuktha R., Gopi A., Robert V., Pugazhenthi G., Loganathan S., Valapa R.B. (2024). Poly(lactic acid)/amine-grafted mesoporous silica-based composite for food packaging application. Int. J. Biol. Macromol..

[115] Du L., Huang X., Li Z., Qin Z., Zhang N., Zhai X., Shi J., Zhang J., Shen T., Zhang R. (2025). Application of smart packaging in fruit and vegetable preservation: A review. Foods.

[116] Palanisamy S., Selvaraju G.D., Selvakesavan R.K., Venkatachalam S., Bharathi D., Lee J. (2024). Unlocking sustainable solutions: Nanocellulose innovations for enhancing the shelf life of fruits and vegetables. Int. J. Biol. Macromol..

[117] Mavai S., Bains A., Sridhar K., Rashid S., Elossaily G.M., Ali N., Chawla P., Sharma M. (2024). Formulation and application of poly lactic acid, gum, and cellulose-based ternary bioplastic for smart food packaging: A review. Int. J. Biol. Macromol..

[118] Jiang Z., Ngai T. (2022). Recent advances in chemically modified cellulose and its derivatives for food packaging applications: A review. Polymers.

[119] Ezati P., Rhim J.W. (2020). pH-responsive pectin-based multifunctional films incorporated with curcumin and sulfur nanoparticles. Carbohydr. Polym..

[120] İbrahim Ş.E.N. (2024). Preparation and characterization of Leonardite and boric acid-added polylactic acid films for food packaging applications. Ind. Crops Prod..

[121] Sultana A., Kathuria A., Gaikwad K.K. (2022). Metal–organic frameworks for active food packaging. A review. Environ. Chem. Lett..

[122] Fu Y., Yang D., Chen Y., Shi J., Zhang X., Hao Y., Zhang Z., Sun Y., Zhang J. (2023). MOF-Based Active Packaging Materials for Extending Post-Harvest Shelf-Life of Fruits and Vegetables. Materials.

[123] Khan A., Riahi Z., Hong S.J., Kim J.Y., Rhim J.W., Min S.C. (2025). Cellulose nanofiber/gelatin-based multifunctional ethylene scavenging active packaging films integrated with TiO2-functionalized Bi (III) metal-organic frameworks. Food Packag. Shelf Life.

[124] Hong S.J., Riahi Z., Khan A., Lee J.G., Kim B.Y., Min S.C., Shin G.H., Kim J.T. (2025). Active packaging film utilizing cerium metal-organic frameworks doped with titanium dioxide as ethylene scavengers for postharvest ripening control of avocados. Food Chem..

[125] Imran A., Ahmed F., Ali Y.A., Naseer M.S., Sharma K., Bisht I.S., Alawadi A.H., Shehzadi U., Islam F., Shah M.A. (2025). A comprehensive review on carbon dot synthesis and food applications. J. Agric. Food Res..

[126] Wu Y., Li W., Feng Y., Shi J. (2025). Carbon Dots as Multifunctional Nanofillers in Sustainable Food Packaging: A Comprehensive Review. Foods.

[127] Nguyen D.H.H., El-Ramady H., Prokisch J. (2025). Food safety aspects of carbon dots: A review. Environ. Chem. Lett..

[128] Priyadarshi R., Uzun S., Rhim J.W. (2024). Edible coating using carbon quantum dots for fresh produce preservation: A review of safety perspectives. Adv. Colloid Interface Sci..

[129] Singh A.K., Itkor P., Lee M., Saenjaiban A., Lee Y.S. (2024). Synergistic Integration of Carbon Quantum Dots in Biopolymer Matrices: An Overview of Current Advancements in Antioxidant and Antimicrobial Active Packaging. Molecules.

[130] Riahi Z., Khan A., Rhim J.W., Shin G.H., Kim J.T. (2025). Red pepper waste-derived carbon dots incorporated sodium alginate/gelatin composite films for bioactive fruit preservation. Int. J. Biol. Macromol..

[131] Riahi Z., Khan A., Ebrahimi M., Rhim J.W., Shin G.H., Kim J.T. (2025). Exploring Sustainable Carbon Dots as UV-Blocking Agents for Food Preservation. Compr. Rev. Food. Sci. Food Saf..

[132] Afshar M.B., Marjani A.P., Balkanloo P.G. (2024). Introducing graphene quantum dots in decomposable wheat starch-gelatin based nano-biofilms. Sci. Rep..

[133] Jiang W., Zhou X., Yuan X., Zhang L., Xiao X., Zhu J., Cheng W. (2025). Multifunctional Metal–Organic Frameworks for Enhancing Food Safety and Quality: A Comprehensive Review. Foods.

[134] Collins J., Yang L., Dong X., Sun Y.P. (2025). Antimicrobial properties of carbon “quantum” dots for food safety applications. J. Nanopart. Res..

[135] Kowalczyk M., Domaradzki P., Skałecki P., Kaliniak-Dziura A., Stanek P., Teter A., Grenda T., Florek M. (2024). Use of sustainable packaging materials for fresh beef vacuum packaging application and product assessment using physicochemical means. Meat Sci..

[136] Yetgin S., Ağırsaygın M., Yazgan İ. (2025). Smart Food Packaging Films Based on a Poly(lactic acid), Nanomaterials, and a pH Sensitive Dye. Processes.

[137] Chaudhary V., Gaur P., Rustagi S. (2024). Sensors, society, and sustainability. Sustain. Mater. Technol..

[138] Fan L., Chen Y., Zeng Y., Yu Z., Dong Y., Li D., Zhang C., Ye C. (2024). Application of visual intelligent labels in the assessment of meat freshness. Food Chem..

[139] Talukder S., Mendiratta S.K., Kumar R.R., Agrawal R.K., Soni A., Luke A., Chand S. (2020). Jamun fruit (Syzgium cumini) skin extract–based indicator for monitoring chicken patties quality during storage. J. Food Sci. Technol..

[140] Wahab S.N., Othman N., Sayuti N.M., Mohaini M.L., Atan R. (2025). Assessing the growth and trends of smart packaging: A case of food distribution. J. Inf. Technol. Manag..

[141] Kalpana S., Priyadarshini S.R., Leena M.M., Moses J.A., Anandharamakrishnan C. (2019). Intelligent packaging: Trends and applications in food systems. Trends Food Sci. Technol..

[142] Shukla V., Kandeepan G., Vishnuraj M.R., Soni A. (2016). Anthocyanins-based indicator sensor for intelligent packaging application. Agric. Res..

[143] Thirupathi Vasuki M., Kadirvel V., Pejavara Narayana G. (2023). Smart packaging—An overview of concepts and applications in various food industries. Food Bioeng..

[144] Kabadurmus O., Kayikci Y., Demir S., Koc B. (2023). A data-driven decision support system with smart packaging in grocery store supply chains during outbreaks. Socio-Econ. Plan. Sci..

[145] Stoica M., Bichescu C.I., Crețu C.-M., Dragomir M., Ivan A.S., Podaru G.M., Stoica D., Stuparu-Crețu M. (2024). Review of Bio-Based Biodegradable Polymers: Smart Solutions for Sustainable Food Packaging. Foods.

[146] Escursell S., Llorach-Massana P., Roncero M.B. (2021). Sustainability in e-commerce packaging: A review. J. Clean. Prod..

[147] Sethunga M., Gunathilake K.D.P.P., Ranaweera K.K.D.S., Munaweera I. (2024). Antimicrobial and antioxidative electrospun cellulose acetate–essential oil nanofibrous membranes for active food packaging. Innov. Food Sci. Emerg. Technol..

[148] Yan M.R., Hsieh S., Ricacho N. (2022). Innovative food packaging, food quality and safety, and consumer perspectives. Processes.

[149] Masamba M. (2024). Impact of Food Packaging Materials on the Shelf-Life and Quality of Packaged Food Products. Int. J. Food Sci..

[150] Ahmed W., Haque A., Mohibbullah M., Khan S.I., Islam M.A., Mondal H.T., Ahmmed R. (2022). A review on active packaging for quality and safety of foods: Current trends, applications, prospects and challenges. Food Packag. Shelf Life.

[151] Mkhari T., Adeyemi J.O., Fawole O.A. (2025). Recent Advances in the Fabrication of Intelligent Packaging for Food Preservation: A Review. Processes.

[152] Srivastava V., Singh S., Das D. Environmental Impact Assessment of Active Biocomposite Packaging and Comparison with Conventional Packaging for Food Application. Proceedings of the DS 130: Proceedings of NordDesign 2024.

[153] Bala A., Laso J., Abejón R., Margallo M., Fullana-i-Palmer P., Aldaco R. (2020). Environmental assessment of the food packaging waste management system in Spain: Understanding the present to improve the future. Sci. Total Environ..

[154] Arfelli F., Roguszewska M., Torta G., Iurlo M., Cespi D., Ciacci L., Passarini F. (2024). Environmental impacts of food packaging: Is it all a matter of raw materials?. Sustain. Prod. Consum..

[155] Li Z.C., Su M.Y., Yuan X.Y., Lv H.Q., Feng R., Wu L.J., Gao X.P., An Y.X., Li Z.W., Li M.Y. (2024). Green fabrication of modified lignin/zeolite/chitosan-based composite membranes for preservation of perishable foods. Food Chem..

[156] Kim T.I., Lee S.J., Chathuranga K., Lee J.S., Kim M.H., Park W.H. (2024). Multifunctional and edible egg white/amylose–tannin bilayer film for perishable fruit preservation. Int. J. Biol. Macromol..

[157] Li Y., Ying Y., Zhou Y., Ge Y., Yuan C., Wu C., Hu Y. (2019). A pH-indicating intelligent packaging composed of chitosan–purple potato extractions strengthened by surface-deacetylated chitin nanofibers. Int. J. Biol. Macromol..

[158] da Silva Ponciano C., Gonzaga F.C., de Oliveira C.P. (2025). Smart packaging based on chitosan acting as indicators of changes in food: A technological prospecting review. Int. J. Biol. Macromol..

[159] Jayasinghe C.V.L., Jayatilake S., Rajapakse H.U.K.D.Z., Gunarathne H.M.N.R., Wanigasinghe H.G. (2024). Consumer perceptions of smart packaging technologies for food. Intelligent Packaging.

[160] Boukid F. (2022). Smart food packaging: An umbrella review of scientific publications. Coatings.

[161] De Jong A.R., Boumans H., Slaghek T., Van Veen J., Rijk R., Van Zandvoort M. (2005). Active and intelligent packaging for food: Is it the future?. Food Addit. Contam..

[162] Prasad P., Kochhar A. (2014). Active packaging in food industry: A review. J. Environ. Sci. Toxicol. Food Technol..

[163] Andrade M.A., Barbosa C.H., Ribero-Santos R., Tomé S., Fernando A.L., Silva A.S., Vilarinho F. (2025). Emerging Trends in Active Packaging for Food: A Six-Year Review. Foods.

[164] Sohail M., Sun D.W., Zhu Z. (2018). Recent developments in intelligent packaging for enhancing food quality and safety. Crit. Rev. Food Sci. Nutr..

[165] Bhutta M.N.M., Ahmad M. (2021). Secure identification, traceability and real-time tracking of agricultural food supply during transportation using internet of things. IEEE Access.

[166] Stramarkou M., Boukouvalas C., Fragkouli D.N., Tsamis C., Krokida M. (2025). Evaluating the Sustainability of Tetra Pak Smart Packaging Through Life Cycle and Economic Analysis. Sustainability.

[167] Deepika L.K., Gaikwad K.K. (2023). Carbon dots for food packaging applications. Sustain. Food Technol..

[168] Kaya M., Akdaşçi E., Eker F., Bechelany M., Karav S. (2025). Recent Advances of Silver Nanoparticles in Wound Healing: Evaluation of In Vivo and In Vitro Studies. Int. J. Mol. Sci..

[169] Fu B., Liu Q., Liu M., Chen X., Lin H., Zheng Z., Zhu J., Dai C., Dong X., Yang D.P. (2022). Carbon dots enhanced gelatin/chitosan bio-nanocomposite packaging film for perishable foods. Chin. Chem. Lett..

[170] Riahi Z., Hong S.J., Rhim J.W., Shin G.H., Kim J.T. (2023). High-performance multifunctional gelatin-based films engineered with metal-organic frameworks for active food packaging applications. Food Hydrocoll..

[171] Lai W.F. (2022). Design of Polymeric Films for Antioxidant Active Food Packaging. Int. J. Mol. Sci..

[172] Carrasco S., Amaro-Gahete J., Espinosa E., Benítez A., Romero-Salguero F.J., Rodríguez A. (2025). Engineering PVA-CNF-MOF Composite Films for Active Packaging: Enhancing Mechanical Strength, Barrier Performance, and Stability for Fresh Produce Preservation. Molecules.

[173] Muthu A., Nguyen D.H.H., Neji C., Törős G., Ferroudj A., Atieh R., Prokisch J., El-Ramady H., Béni Á. (2025). Nanomaterials for Smart and Sustainable Food Packaging: Nano-Sensing Mechanisms, and Regulatory Perspectives. Foods.

[174] Nayak A., Dutta D. (2023). A comprehensive review on CRISPR and artificial intelligence based emerging food packaging technology to ensure “safe food”. Sustain. Food Technol..

[175] Galanakis C.M. (2020). The food systems in the era of the coronavirus (COVID-19) pandemic crisis. Foods.

[176] Davidescu M.-A., Pânzaru C., Mădescu B.-M., Radu-Rusu R.-M., Doliș M.G., Simeanu C., Usturoi A., Ciobanu A., Creangă Ș. (2024). Genetic Diversity and Phylogenetic Analysis of the Endangered Transylvanian Pinzgau Cattle: A Key Resource for Biodiversity Conservation and the Sustainability of Livestock Production. Agriculture.

[177] Li H., Wang B. (2023). Green packaging materials design and efficient packaging with Internet of Things. Sustain. Energy Technol. Assess..

[178] Sharma S., Gahlawat V.K., Rahul K., Mor R.S., Malik M. (2021). Sustainable innovations in the food industry through artificial intelligence and big data analytics. Logistics.

[179] Purnama S., Sejati W. (2023). Internet of things, big data, and artificial intelligence in the food and agriculture sector. Int. J. Artif. Intell..

[180] Paliwoda B., Górna J., Biegańska M., Wójcicki K. (2023). Application of industrial internet of things (IIoT) in the packaging industry in Poland. LogForum.

[181] AlZubi A.A., Galyna K. (2023). Artificial intelligence and internet of things for sustainable farming and smart agriculture. IEEE Access.

[182] Stramarkou M., Boukouvalas C., Koskinakis S.E., Serifi O., Bekiris V., Tsamis C., Krokida M. (2022). Life Cycle Assessment and Preliminary Cost Evaluation of a Smart Packaging System. Sustainability.

[183] Hamad A., Tayel A. (2025). Food 2050 Concept: Trends That Shape the Future of Food. J. Future Food.

[184] Panou A., Lazaridis D.G., Karabagias I.K. (2025). Application of Smart Packaging on the Preservation of Different Types of Perishable Fruits. Foods.

